# Copper Induces Protein Aggregation, a Toxic Process Compensated by Molecular Chaperones

**DOI:** 10.1128/mbio.03251-21

**Published:** 2022-03-15

**Authors:** Lisa Zuily, Nora Lahrach, Rosi Fassler, Olivier Genest, Peter Faller, Olivier Sénèque, Yann Denis, Marie-Pierre Castanié-Cornet, Pierre Genevaux, Ursula Jakob, Dana Reichmann, Marie-Thérèse Giudici-Orticoni, Marianne Ilbert

**Affiliations:** a Aix-Marseille Université, CNRS, BIP, UMR 7281, IMM, Marseille, France; b Department of Biological Chemistry, The Alexander Silberman Institute of Life Sciences, Safra Campus Givat Ram, The Hebrew University of Jerusalem, Jerusalem, Israel; c Biometals and Biology Chemistry, Institut de Chimie (CNRS UMR7177), Université de Strasbourg, Strasbourg, France; d Université Grenoble Alpes, CNRS, CEA, IRIG/DIESE, LCBM (UMR 5249), Grenoble, France; e Plateforme Transcriptome, Aix-Marseille Université, CNRS, IMM-FR3479, Marseille, France; f Laboratoire de Microbiologie et Génétique Moléculaires, Centre de Biologie Intégrative, Université de Toulouse, CNRS, UPS, Toulouse, France; g Department of Molecular, Cellular and Developmental Biology, University of Michigan, Ann Arbor, Michigan, USA; University of Arizona

**Keywords:** copper stress, molecular chaperone, protein aggregation, copper homeostasis, *Escherichia coli*, DnaK, trigger factor, proteostasis, copper tolerance, heat shock, stress response

## Abstract

Copper is well known for its antimicrobial and antiviral properties. Under aerobic conditions, copper toxicity relies in part on the production of reactive oxygen species (ROS), especially in the periplasmic compartment. However, copper is significantly more toxic under anaerobic conditions, in which ROS cannot be produced. This toxicity has been proposed to arise from the inactivation of proteins through mismetallations. Here, using the bacterium Escherichia coli, we discovered that copper treatment under anaerobic conditions leads to a significant increase in protein aggregation. *In vitro* experiments using E. coli lysates and tightly controlled redox conditions confirmed that treatment with Cu^+^ under anaerobic conditions leads to severe ROS-independent protein aggregation. Proteomic analysis of aggregated proteins revealed an enrichment of cysteine- and histidine-containing proteins in the Cu^+^-treated samples, suggesting that nonspecific interactions of Cu^+^ with these residues are likely responsible for the observed protein aggregation. In addition, E. coli strains lacking the cytosolic chaperone DnaK or trigger factor are highly sensitive to copper stress. These results reveal that bacteria rely on these chaperone systems to protect themselves against Cu-mediated protein aggregation and further support our finding that Cu toxicity is related to Cu-induced protein aggregation. Overall, our work provides new insights into the mechanism of Cu toxicity and the defense mechanisms that bacteria employ to survive.

## INTRODUCTION

Copper (Cu) ions have been exploited for their antibacterial properties since antiquity (1, 2). In biological systems, Cu enters the cell either by passive diffusion or through yet to be identified metal importers (3). Cu is typically found in two redox states, Cu^+^ [Cu(I)] and Cu^2+^ [Cu(II)]. While Cu^+^ predominates in the highly reducing environment of the bacterial cytoplasm, both redox forms are present in the oxidizing environment of the periplasm. The predominance of Cu^+^ in the cytoplasm is in large part due to the presence of reduced glutathione (GSH), one of the most abundant thiol-containing tripeptides in most biological systems (>5 mM in Escherichia coli cytoplasm) (4, 5). Glutathione rapidly reduces any “free” Cu^2+^ to Cu^+^ and by complexing Cu^+^ stabilizes the Cu^+^ state (6). In the periplasm, Cu^2+^ oxidizes cellular macromolecules, gets reduced to Cu^+^, and then readily reoxidizes through interactions with O_2_ and H_2_O_2_ (i.e., the Fenton reaction). The hydroxyl radicals that are formed in this process are highly reactive and cause further oxidative damage to cells and organisms (7, 8). Not surprisingly, the observed Cu toxicity has therefore long been attributed to the Cu^+^-mediated generation of reactive oxygen species (ROS) and the accompanying oxidative damage to cellular macromolecules (1, 9). Further support for this model came from experiments that showed copper treatment leads to the overexpression of genes involved in oxidative stress response as well as to an increase in lipid peroxidation (10, 11).

However, an alternative mechanism for Cu toxicity has also been proposed. Imlay and colleagues observed efficient Cu-mediated killing of E. coli under anaerobic conditions, eliminating ROS as a possible culprit (12, 13). *In vivo* and *in vitro* studies demonstrated that Cu^+^ inactivates essential iron-sulfur cluster enzymes in an ROS-independent manner (13–15). In other organisms, copper toxicity has also been linked to the more general inactivation of cellular pathways, such as the central carbon metabolism (16, 17) or the nucleotide synthesis pathway (18). In addition to mismetallation, it has also been proposed that Cu ions might trigger protein misfolding and aggregation (17, 19–21). However, these studies were performed under aerobic conditions, making it difficult to assess whether the observed effects are elicited directly by either Cu^2+^ or Cu^+^ or indirectly through ROS formation. The aim of this study was to shed light on the effects of Cu^+^ on protein folding and stability by working under strictly anaerobic conditions both *in vitro* and *in vivo*. We demonstrate *in vivo* that CuSO_4_ treatment under anaerobic conditions causes substantially more protein unfolding and protein aggregation than CuSO_4_ treatment under ROS-producing aerobic conditions. This increased protein aggregation is likely due to a significantly more pronounced accumulation of copper in the bacterial cytoplasm under anaerobic conditions. Consistent with these results, we discovered that the bacterial chaperones DnaK/DnaJ/GrpE (DnaKJE) and trigger factor efficiently protect cells against copper toxicity, particularly under anaerobic conditions. These studies reveal one of the main mechanisms of Cu toxicity under anaerobic conditions.

## RESULTS

### Copper induces significant protein aggregation under anaerobic conditions *in vivo*.

**FIG 1:**
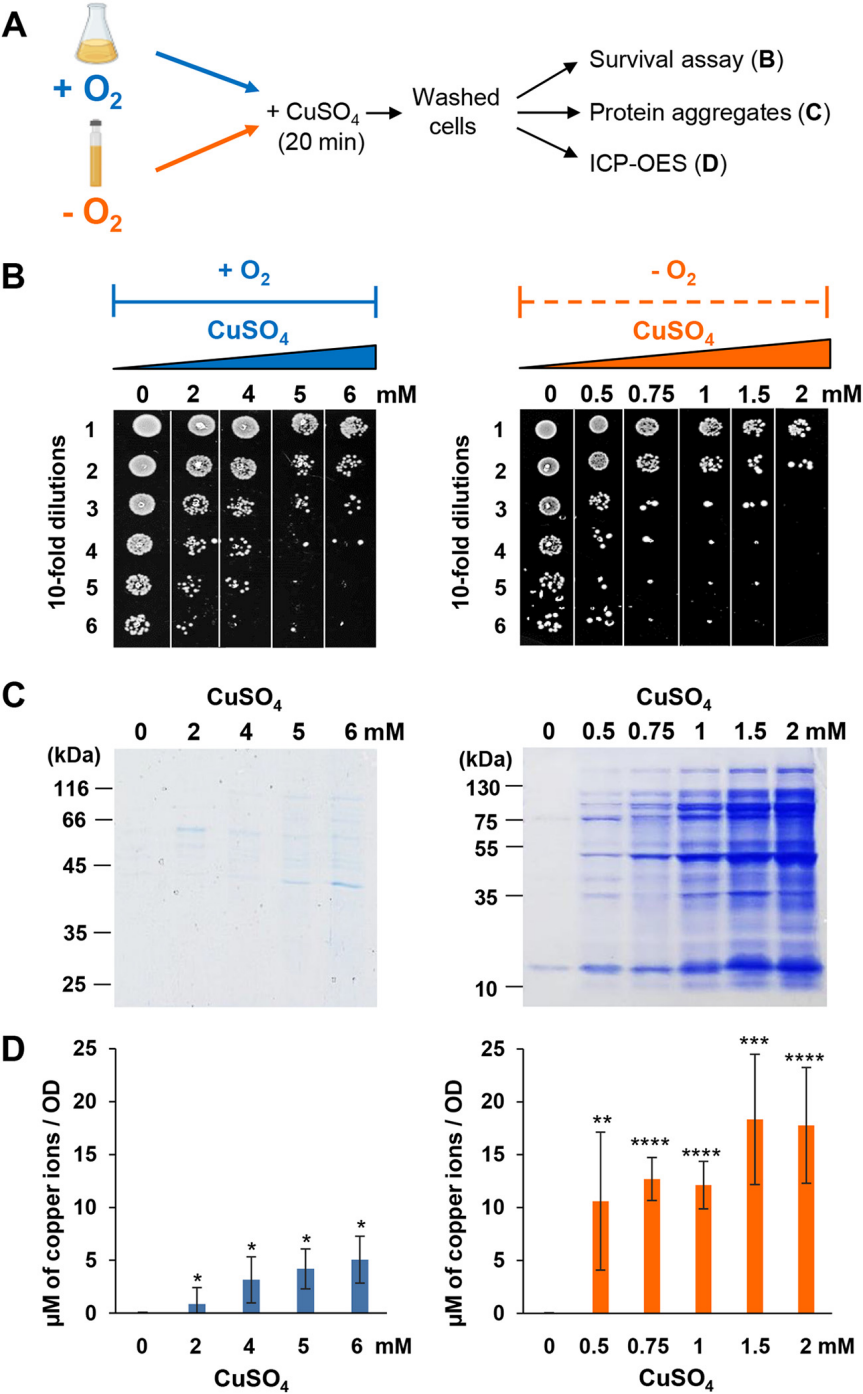
CuSO_4_ induces protein unfolding under anaerobic conditions *in vivo*. (A) E. coli cells were grown until they reached an OD_600_ of 0.7 and exposed to different concentrations of CuSO_4_ for 20 min at 37°C under aerobic (in blue) or anaerobic (in orange) conditions. Cells were washed several times to remove copper excess. They were subsequently analyzed by three different assays. (B) Three microliters of 10-fold serial dilutions was spotted onto LB agar plates in an aerobic (left panel) or anaerobic (right panel) atmosphere and incubated at 37°C. The plates are representative of at least three independent experiments. (C) Cells were lysed and after different centrifugation steps, aggregated proteins were isolated and loaded on 12% SDS-PAGE; the gels are representative of at least three independent experiments. (D) The amount of intracellular Cu content was measured by ICP-OES. Error bars represent standard deviations (SD) from triplicate experiments. Statistical analyses were performed using an unpaired two-tailed *t* test (*, *P* < 0,05; **, *P* < 0,01; ***, *P* < 0.001; ****, *P* < 0.0001), using their respective results from 0 mM treated cells as the comparative value.

The presence of excess Cu^2+^ in the growth medium has been shown to trigger protein aggregation in bacteria grown under aerobic conditions (22). However, it is difficult to untangle whether protein aggregation is caused by Cu^2+^, Cu-mediated ROS production, or directly by Cu^+^. We therefore decided to monitor growth in the presence of copper either under anaerobic conditions to avoid the Cu-induced Fenton reaction or under aerobic conditions. In addition, we evaluated two other parameters: we quantified the amount of intracellular copper content via inductively coupled plasma optical emission spectrometry (ICP-OES) and analyzed intracellular protein aggregation by SDS-PAGE. For all of these experiments, we exposed cells grown under either anaerobic or aerobic conditions to various CuSO_4_ concentrations for 20 min (Fig. 1A). We then washed the cells to remove any adventitiously associated copper and serially diluted the bacteria onto LB agar plates to determine cell survival (Fig. 1A and B). As expected, we found that copper is highly toxic to bacteria, yet the copper toxicity was significantly more pronounced under anaerobic conditions. While we observed a significant 4-log decrease in cell survival in the presence of 5 mM CuSO_4_ under aerobic conditions, 0.75 mM CuSO_4_ was sufficient to cause the same effects under anaerobic conditions (Fig. 1B). We also found massive differences in the degree of protein aggregation between aerobic and anaerobic copper stress (Fig. 1C), concomitant with substantial differences in their intracellular copper contents (Fig. 1D). E. coli cells, stressed with copper under anaerobic conditions, accumulated higher levels of intracellular copper and revealed a disproportionally high accumulation of insoluble protein aggregates (Fig. 1C and D). These results revealed that E. coli is sensitive toward copper-induced protein aggregation under anaerobic growth.

### Cu^+^ ions act as efficient protein aggregating reagents in a ROS-independent manner *in vitro*.

**FIG 2:**
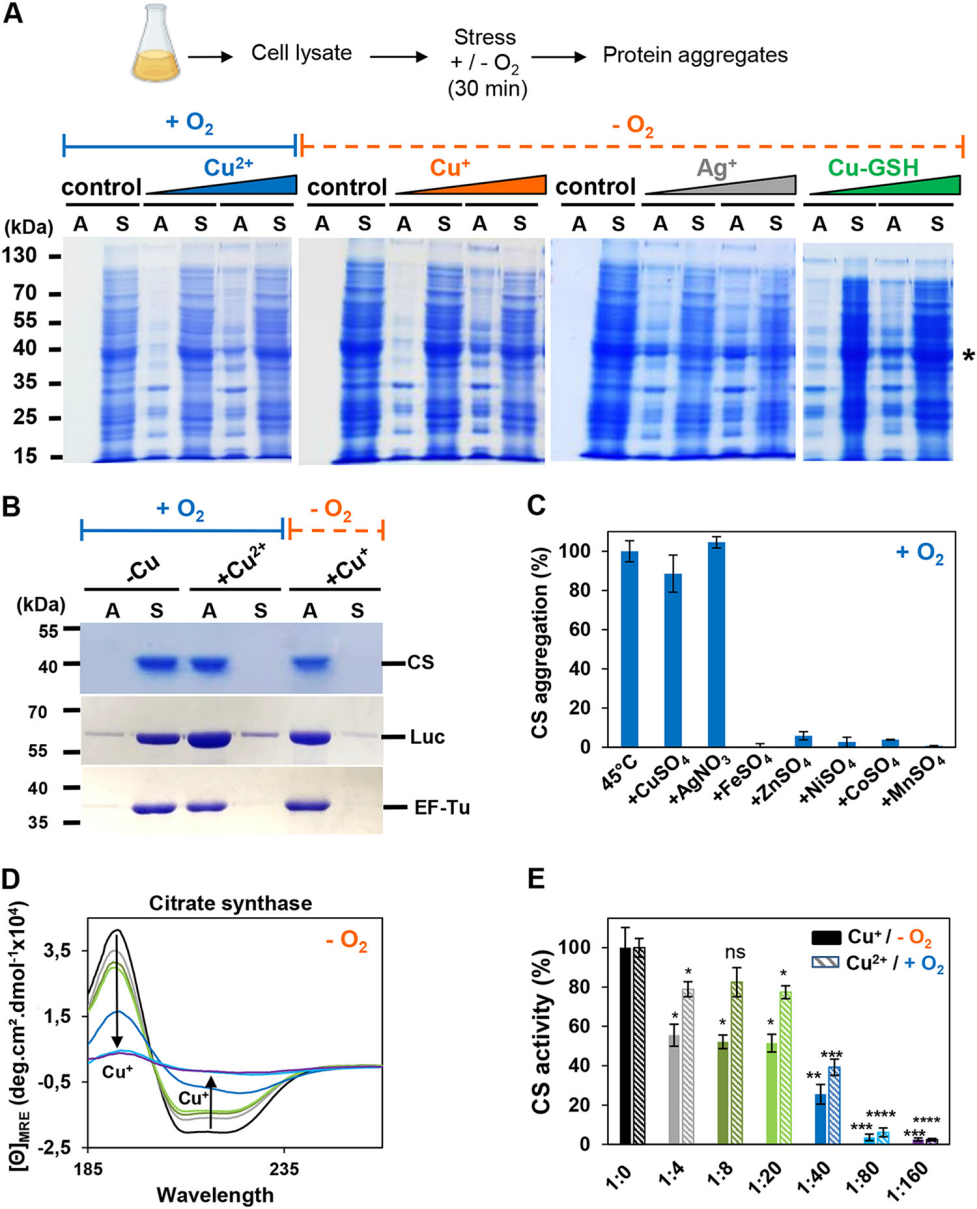
*In vitro* inactivation, unfolding, and aggregation of proteins by Cu^+^ or Cu^2+^. (A) A 1-mg/mL concentration of E. coli lysate was incubated with or without 100 and 500 μM Cu^2+^ under aerobic conditions and Cu^+^, Ag^+^, or Cu-GSH under anaerobic conditions. Control samples correspond to the absence of copper; in the case of Cu^+^ treatment, the same amount of acetonitrile was added in the control samples. Aggregates (A) and soluble proteins (S) were separated by centrifugation and analyzed by 12% SDS-PAGE. An asterisk indicates EF-Tu protein identified by mass spectrometry. This experiment was performed in triplicate, and a representative result is shown. (B) A 2 μM concentration of citrate synthase (CS), luciferase (Luc), or EF-Tu was incubated for 30 min at 30°C in the absence of metal ions or in the presence of 80-fold molar excess of Cu^2+^ (under aerobic conditions) or Cu^+^ (under anaerobic conditions). Aggregates (A) and soluble proteins (S) were separated by centrifugation and analyzed by 12% SDS-PAGE. This experiment was performed in triplicate, and representative results are shown. (C) Aggregation of CS (2 μM) was measured by light scattering (360 nm) at 30°C, and end points were taken after 15 min of incubation with 80-fold molar excess of different metals under aerobic conditions. A value obtained at 30°C in the absence of metal ions was defined as 0%, and a value obtained after 15 min at 45°C corresponds to 100% of CS aggregation. Error bars represent standard deviation (SD). (D) Far-UV CD spectra of 2.5 μM CS treated without metal (black) or with a 4-fold (gray), 8-fold (dark green), 20-fold (light green), 40-fold (dark blue), 80-fold (light blue), or 160-fold (purple) molar excess of Cu^+^ (under anaerobic conditions) were recorded at 25°C. A representative result from several experiments is shown. (E) A 1.5 nM concentration of CS was incubated without metal (black) or with a 4-fold (gray), 8-fold (dark green), 20-fold (light green), 40-fold (dark blue), 80-fold (light blue), or 160-fold (purple) molar excess of Cu^+^ (under anaerobic conditions) or Cu^2+^ (same color code, with hatching, under aerobic conditions), at 25°C for 30 min, and CS-specific activity was monitored. CS-specific activity obtained without Cu addition represents 100% of CS activity. Error bars represent standard deviation (SD). Statistical analyses were performed with an unpaired two-tailed *t* test (*, *P* < 0.05; **, *P* < 0.01), using CS without metal as a comparative value.

To further investigate the differences observed between cells exposed to copper in the presence or absence of oxygen, we performed *in vitro* studies using cellular extracts. We reasoned that by using *ex vivo* assays, we will be able to tightly control the Cu redox status and directly investigate the impact of Cu^2+^ and Cu^+^ ions on the stability of proteins. Therefore, we prepared soluble protein extracts of E. coli cells and exposed them to different Cu treatments, which we will refer to as “Cu^+^ stress” or “Cu^2+^ stress.” Cu^+^ stress [i.e., addition of tetrakis(acetonitrile) copper(I) hexafluorophosphate under strictly controlled anaerobic conditions to prevent any ROS production] mimics the accumulation of intracellular cytoplasmic copper under anaerobic conditions. In contrast, Cu^2+^ stress (i.e., addition of CuSO_4_ under aerobic conditions) represents reactions that typically occur when cells grow under aerobic conditions in the presence of a high level of CuSO_4_. We then treated the cell lysates with previously established *in vitro* concentrations (19, 20) of 100 or 500 μM Cu^2+^ stress under aerobic conditions or Cu^+^ stress under anaerobic conditions for 30 min, separated the soluble and insoluble proteins by centrifugation, and monitored the proteins by reducing SDS-PAGE (Fig. 2A). To our surprise, we did not observe any significant differences in the extent of protein aggregation between Cu^+^ stress- or Cu^2+^ stress-exposed cell lysates, suggesting that the differences observed *in vivo* are not due to the Cu redox state but might be due to the higher levels of intracellular copper concentrations that accumulate under anaerobic conditions (Fig. 1C and D). Moreover, these results clearly show that Cu^+^-induced protein aggregation is not the result of oxidative damage but must be triggered directly by Cu^+^. To independently confirm this conclusion, we treated the cell lysates with AgNO_3_ under anaerobic conditions. Ag^+^ has a similar coordination chemistry to Cu^+^, yet in contrast to Cu^+^, Ag^+^ is not redox active and does not catalyze the Fenton reaction. Like Cu^+^, we found that Ag^+^ induces the aggregation of numerous E. coli proteins (Fig. 2A).

The bacterial cytosol contains high levels of reduced glutathione (GSH), which is able to bind to Cu^+^ and hence protects cytoplasmic proteins from copper-induced damage (13, 21). To test the impact of GSH on protein-induced aggregation *in vitro*, we incubated CuSO_4_ (i.e., Cu^2+^) with physiological concentrations of GSH (5 mM). Under these conditions, Cu^2+^ is immediately reduced to Cu^+^ and tightly bound by the tripeptide (from here on “Cu-GSH”) (23). To prevent any Cu^+^ or cysteine oxidation by dioxygen or ROS formation, we conducted these experiments also under fully anaerobic conditions. Incubation of cell lysates with Cu-GSH for 30 min under the conditions of our experiment caused substantial protein aggregation (Fig. 2A). This observation is consistent with the above result (Fig. 1) showing the formation of aggregates *in vivo* induced by copper.

However, GSH is known *in vivo* to protect cells against intracellular copper toxicity (21). To better understand the role played by GSH *in vivo* under copper stress (aerobic versus anaerobic), we measured cell viability in the presence of copper of two mutant strains affected in their ability to produce GSH. These strains lack either *gshA* (encoding a γ-glutamylcysteine synthetase) or *gshB* (encoding a glutathione synthetase). After exposure to copper stress, the two mutant strains exhibited a significant decrease in cell survival compared to the wild-type (WT) strain under anaerobic conditions (see Fig. S1 in the supplemental material). This result confirms that GSH protects cells against copper damage. The effect was observed mainly under anaerobic conditions, suggesting that the high level of intracellular copper measured (Fig. 1B) accumulates in the cytosol and that cell death under this condition is due to cytoplasmic damage. Copper complexation to GSH could limit copper binding to proteins; however, a slight cytoplasmic increase in copper could modify the equilibrium, and depending on Cu^+^ affinity to some proteins, its binding could induce protein aggregation, as observed *in vivo* (Fig. 1B) and *in vitro* (Fig. 2A).

10.1128/mbio.03251-21.1 FIG S1 Importance of *gshA* or *gshB* for cell survival in the presence of copper under aerobic versus anaerobic conditions. Cells (WT or Δ*gshA* and Δ*gshB* mutants from the KEIO collection) were grown at 37°C until they reached an OD_600_ of 0.7. Cells were treated with increasing concentrations of CuSO_4_, anaerobically or aerobically for 20 min at 37°C, as depicted in Fig. 4A. Cells were serial diluted in LB, spotted on LB plates, and then incubated for 16 h. Plates are representatives of at least three experiments. Download FIG S1, JPG file, 0.1 MB. Copyright © 2022 Zuily et al. 2022 Zuily et al. https://creativecommons.org/licenses/by/4.0/ This content is distributed under the terms of the Creative Commons Attribution 4.0 International license.

### Cu^+^ ions inactivate and unfold purified proteins in a concentration-dependent manner.

To obtain more detailed insights into the impact of Cu^+^ and Cu^2+^ on protein stability, we performed similar experiments but used purified proteins instead. We tested citrate synthase (CS) and firefly luciferase, two well-established aggregation-prone proteins (24), as well as purified EF-Tu from E. coli, which we found to be highly Cu sensitive in cell lysates (see the asterisk in Fig. 2A). As indicated above, we treated the proteins with either Cu^2+^ or Cu^+^ under aerobic or anaerobic conditions, respectively, and monitored protein precipitation after 30 min of incubation. As shown in Fig. 2B, independent of the redox form of copper, all three proteins aggregated within the given time frame of the experiment. Control experiments showed that neither the counterions nor the buffer alone caused any protein aggregation (see Fig. S2 in the supplemental material). Again, we used Ag^+^ to further exclude the involvement of ROS in our Cu^+^-treated samples and found that CS aggregation also occurred in the presence of this non-redox metal (Fig. 2C). Importantly, none of the other metals that we tested, including FeSO_4_, NiCl_2_, ZnSO_4_, and CoCl_2_, caused any significant aggregation of CS (Fig. 2C).

10.1128/mbio.03251-21.2 FIG S2 Cupric and cuprous ions induce CS aggregation independently of the buffer or of the counterion. (A) After incubation of 2 μM CS in the presence or absence of an 80-fold molar excess of Cu^2+^ (under aerobic conditions), Cu^+^ (resuspended in 10% acetonitrile under anaerobic conditions) or AcN (10% acetonitrile) at 30°C for 30 min, aggregates (A) and soluble proteins (S) were separated by centrifugation and analyzed by 12% SDS–PAGE. (B and C) Aggregation of 2 μM CS was measured by light scattering (360 nm) at 30°C. After 15 min of incubation with an 80-fold molar excess of CuSO_4_ or CuCl_2_ in 40 mM MOPS buffer at pH 7.5 (B and C) or in 40 mM HEPES at pH 7.5 (C), absorbance at 360 nm was measured. A value obtained in the absence of metal was defined as 0% aggregation, and a value obtained after 15 min of incubation at 45°C corresponds to 100%. The standard deviation is represented by error bars calculated from at least three independent experiments. Download FIG S2, JPG file, 0.03 MB. Copyright © 2022 Zuily et al. 2022 Zuily et al. https://creativecommons.org/licenses/by/4.0/ This content is distributed under the terms of the Creative Commons Attribution 4.0 International license.

To determine the minimum amount of Cu^+^ required to induce CS aggregation, we monitored CS aggregation by light scattering, which is more sensitive than a solubility test, and used rubber-sealed cuvettes to maintain anaerobic conditions. We found that under our experimental conditions, CS began to aggregate when we reached a CS/Cu^+^ ratio of 1:40 (see Fig. S3A in the supplemental material). To test whether lower Cu^+^ concentrations can change the secondary structure of CS without triggering visible protein aggregation, we monitored far-UV circular dichroism (CD) spectra of Cu^+^-treated CS incubated in rubber-sealed cuvettes (Fig. 2D). We observed that a 4-fold molar excess of Cu^+^ ions to CS is sufficient to cause a significant loss of signal from the initial circular dichroism spectrum, followed by the inactivation of CS (Fig. 2E). We obtained very similar results when we treated CS with Cu^2+^ under aerobic conditions (Fig. 2E; Fig. S3). We concluded from these results that both redox states of Cu cause a loss of protein structure and function *in vitro*.

10.1128/mbio.03251-21.3 FIG S3 Cupric and cuprous ions induce CS unfolding. (A) Aggregation of CS (2 μM) was measured by light scattering (360 nm) at 30°C, and end points were taken after 30 min of incubation with a 4-fold (gray), 8-fold (dark green), 20-fold (light green), 40-fold (dark blue), 80-fold (light blue), or 160-fold (purple) molar excess of Cu^+^ (under anaerobic conditions, plain) or Cu^2+^ (under aerobic conditions, same color code, hatched) at 30°C. The graph shows the mean of three independent experiments; error bars correspond to SD. (B) Far-UV CD spectra of CS were recorded on samples treated without metal (black) or with a 4-fold (gray), 8-fold (dark green), 20-fold (light green), 40-fold (dark blue), 80-fold (light blue), or 160-fold (purple) molar excess of Cu^2+^ (under aerobic conditions). A representative result from several experiments is shown. (C) Values obtained at 220 nm by far-UV CD spectra of CS were plotted for samples treated without metal (black) or with a 4-fold (gray), 8-fold (dark green), 20-fold (light green), 40-fold (dark blue), 80-fold (light blue), or 160-fold (purple) molar excess of Cu^+^ (under anaerobic conditions, plain) or Cu^2+^ (under aerobic conditions, same color code, hatched). Download FIG S3, JPG file, 0.1 MB. Copyright © 2022 Zuily et al. 2022 Zuily et al. https://creativecommons.org/licenses/by/4.0/ This content is distributed under the terms of the Creative Commons Attribution 4.0 International license.

### Identification and comparative analysis of copper-induced protein aggregates.

**FIG 3:**
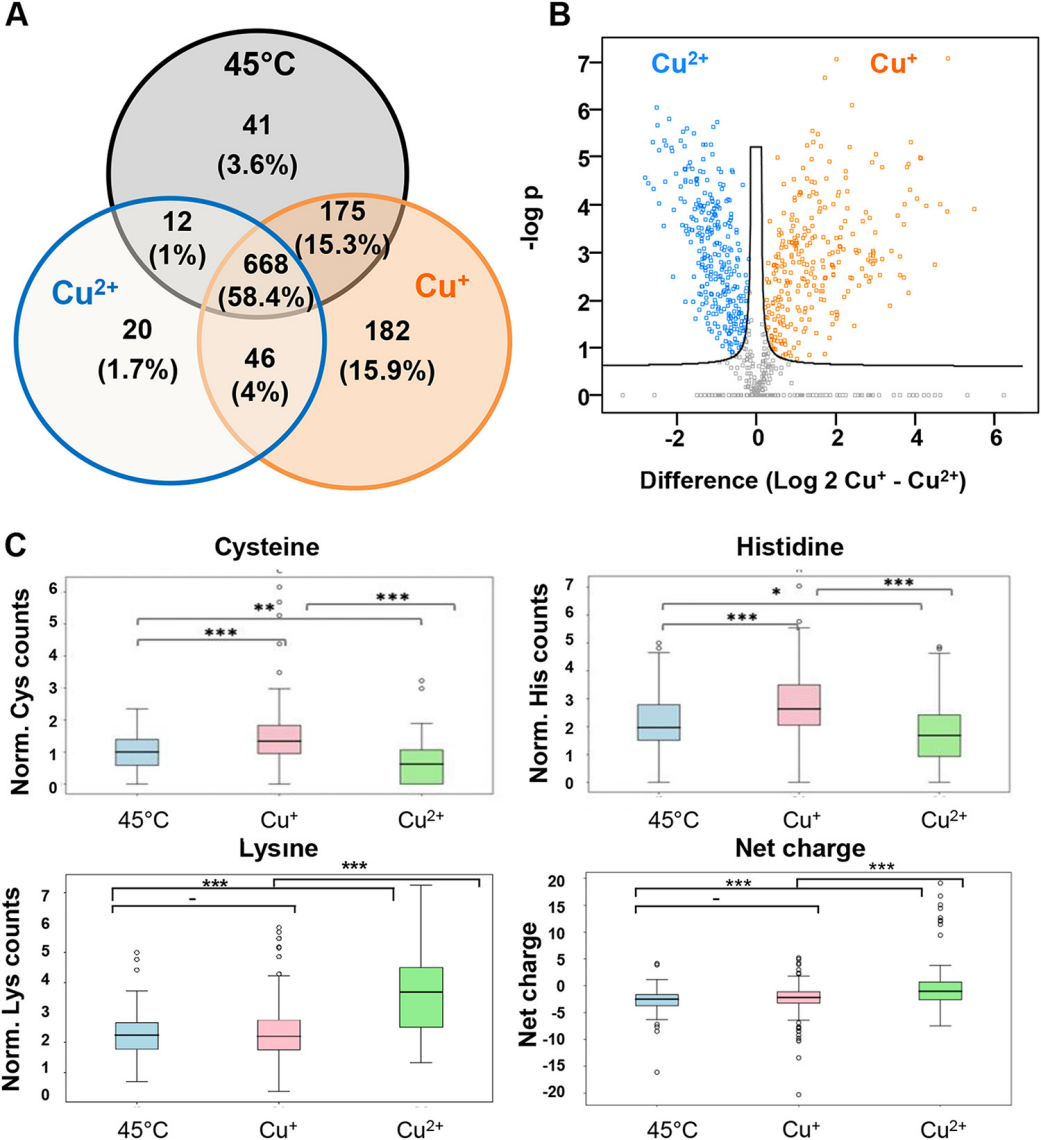
Aggregation-prone proteins and changes in abundance of proteins/residues after different treatments. (A) Venn diagram of identified insoluble proteins by mass spectrometry analysis. Insoluble proteins obtained at 30°C were subtracted from all three sets. The number of proteins identified under each condition is depicted, with the total number of aggregates under all three conditions set to 100%. (B) Volcano plots represent comparative analysis of differential abundance of insoluble proteins obtained after two different treatments. Significantly abundant proteins are colored in either orange (Cu^+^) or blue (Cu^2+^), according to the FDR of 0.05 and a fold change greater than 2. The volcano plot is related to Table S1. (C) Box plots of amino acid propensities (cysteine, histidine, and lysine) or net charge of proteins aggregated following a treatment with either 45°C (blue), Cu^+^ (pink), or Cu^2+^ (green). The features were normalized to the protein length. PASTA 2.0 (26) was used for prediction of protein aggregation and disorder propensity. Instability and amino acid propensities were evaluated by using in-house scripts with Python methods from the website https://biopython.org/DIST/docs/api/Bio.SeqUtils.ProtParam.ProteinAnalysis-class.html#flexibility. Two-tailed Student's *t* tests with similar variance were used to evaluate statistically different features. Significantly different sets are marked according to *P* values of 0.001 (***), 0.01 (**), or 0.05 (*).

We next asked whether proteins differ in their aggregation sensitivity following Cu^2+^ or Cu^+^ stress. To address this issue, we conducted a comparative proteomic analysis of aggregates from lysates treated with 100 μM Cu^+^ under anaerobic conditions or with 100 μM Cu^2+^ under aerobic conditions (Fig. 2A). As controls, we analyzed the protein aggregates from lysates incubated for 30 min at 30 or 45°C. To identify and quantify changes in protein abundance in the different samples, we applied label-free quantification (LFQ) using the MaxQuant software (25). We identified 1,144 proteins across all three treatments that did not appear in the 30°C control treatment (see Table S1 in the supplemental material). As shown in the Venn diagram (Fig. 3A), all three treatments caused the aggregation of a common set of 668 proteins, which comprises about 58% of the aggregated proteome. Many of these proteins have been previously shown to be aggregation sensitive ( (26, 27). Of the remaining 476 aggregation-prone proteins, most proteins (i.e., 403) were sensitive to Cu^+^ stress, and 182 proteins were exclusively found to aggregate upon Cu^+^ treatment. In response to Cu^2+^ treatment, 78 additional proteins aggregated, of which only 20 proteins were uniquely sensitive to Cu^2+^ treatment. The heat treatment resulted in 41 (from 228) distinctly aggregated proteins.

10.1128/mbio.03251-21.8 TABLE S1 List of all identified proteins in the proteomic analysis of aggregates that are induced either by heat shock (45°C) or by treatment with Cu^+^ or Cu^2+^. All of the listed proteins were absent in the background control (aggregates from the untreated sample). Other sections depict proteins used for the Venn diagram as well as proteins used for in depth data analysis. Download Table S1, XLSX file, 0.3 MB. Copyright © 2022 Zuily et al. 2022 Zuily et al. https://creativecommons.org/licenses/by/4.0/ This content is distributed under the terms of the Creative Commons Attribution 4.0 International license.

To quantify stress-specific changes in the abundance of the aggregated proteins, we performed a Student's *t* test analysis and defined the subset of proteins that was significantly enriched under either heat, Cu^2+^, or Cu^+^ treatment (Fig. 3B; see Fig. S4 in the supplemental material). This protein profiling clearly revealed the differential sensitivity of some proteins toward the type of stress treatment. We compiled a data set of proteins that were uniquely sensitive (Fig. 3A) or significantly enriched (Fig. 3B, more than 4-fold change) relative to the other two conditions (Table S1). A functional enrichment analysis did not reveal any statistically significant preference for any specific pathway or biological function in the aggregated proteome in either of the treatments (see Table S2A in the supplemental material). However, we observed the inactivation of key proteins involved in metabolic pathways (for example, enzymes of the central carbon metabolism) that could explain the global impact on cells. We also noticed that metal binding proteins seemed to be more impacted by Cu^+^ than Cu^2+^ treatment, as illustrated by the overrepresentation of zinc binding proteins, iron transport/storage proteins, or oxidoreductases among the Cu^+^-sensitive proteins (Table S2A). These results are in agreement with previous *in vivo* reports, suggesting that Cu^+^ targets metalloproteins (13, 14, 17).

10.1128/mbio.03251-21.4 FIG S4 Comparative analysis of different treatments illustrated by volcano plots. Volcano plots represent comparative analysis of differential abundance of insoluble proteins obtained after two different treatments. Significantly abundant proteins are colored in either black (treatment at 45°C), blue (Cu^2+^) (A), or orange (Cu^+^) (B), according to an FDR of 0.05 and a fold change of greater than 2. The volcano plots are related to Table S1. Download FIG S4, JPG file, 0.1 MB. Copyright © 2022 Zuily et al. 2022 Zuily et al. https://creativecommons.org/licenses/by/4.0/ This content is distributed under the terms of the Creative Commons Attribution 4.0 International license.

10.1128/mbio.03251-21.9 TABLE S2 Bioinformatics analysis of protein aggregates identified by proteomics. (A) Functional annotation of differentially treated insoluble proteins. (B) *t* test comparison of sequence features of insoluble proteins obtained by different treatments. Download Table S2, DOCX file, 0.02 MB. Copyright © 2022 Zuily et al. 2022 Zuily et al. https://creativecommons.org/licenses/by/4.0/ This content is distributed under the terms of the Creative Commons Attribution 4.0 International license.

The next step in our data analysis was to identify the intrinsic properties that make proteins sensitive to copper stress. The strength of our *in vitro* approach using cellular extracts is to avoid modifications of the proteome that would occur *in vivo* in response to copper, such as transcriptional regulation or protein degradation. We compared sequence compositions among our previously selected groups of proteins (Fig. 3C; see Table S1, Table S2B, and Fig. S5 in the supplemental material). This analysis showed that heat treatment affects preferentially larger proteins (i.e., average size of 445 amino acids [aa]) compared to proteins precipitated by Cu^2+^ or Cu^+^ (Fig. S5C). In contrast, Cu^+^-sensitive proteins were found to be slightly enriched in cysteine and histidine residues relative to the Cu^2+^-sensitive proteins (Fig. 3C; Table S2B). As cysteine and histidine residues are known to have a high binding affinity for Cu^+^ at neutral pH (28, 29), this analysis indicates that Cu^+^ might promote protein aggregation by preferentially interacting with these residues. In contrast, Cu^2+^ appears to affect preferentially charged proteins, suggesting that Cu^2+^ might interfere with electrostatic interactions by a mechanism that remain to be determined. Interestingly, the distribution of predicted secondary structural elements was found to be similar in all three protein groups, suggesting that the sequence rather than the structure of proteins defines their sensitivity to unfold upon specific stress. These results demonstrate that although both Cu states can induce protein aggregation *in vitro*, Cu^2+^ and Cu^+^ treatments target different subsets of proteins.

10.1128/mbio.03251-21.5 FIG S5 Box plots of amino acid propensities (A and B), aromaticity, length, and isoelectric point, as well as predicted protein instability (C), of aggregated proteins following a treatment with either 45°C (blue), Cu^+^ (pink), or Cu^2+^ (green). The features were normalized to the protein length. PASTA 2.0 (26) was used for prediction of protein aggregation and disorder propensity. Instability and amino acid propensities were evaluated by using in-house scripts with Python methods from the website https://biopython.org/DIST/docs/api/Bio.SeqUtils.ProtParam.ProteinAnalysis-class.html#flexibility. A two-tailed Student’s *t* test with similar variance was used to evaluate statistically different features. Significantly different sets are marked according to *P* values of 0.001 (***), 0.01 (**), or 0.05 (*). Download FIG S5, JPG file, 0.5 MB. Copyright © 2022 Zuily et al. 2022 Zuily et al. https://creativecommons.org/licenses/by/4.0/ This content is distributed under the terms of the Creative Commons Attribution 4.0 International license.

### Copper induces expression of stress response genes.

**FIG 4:**
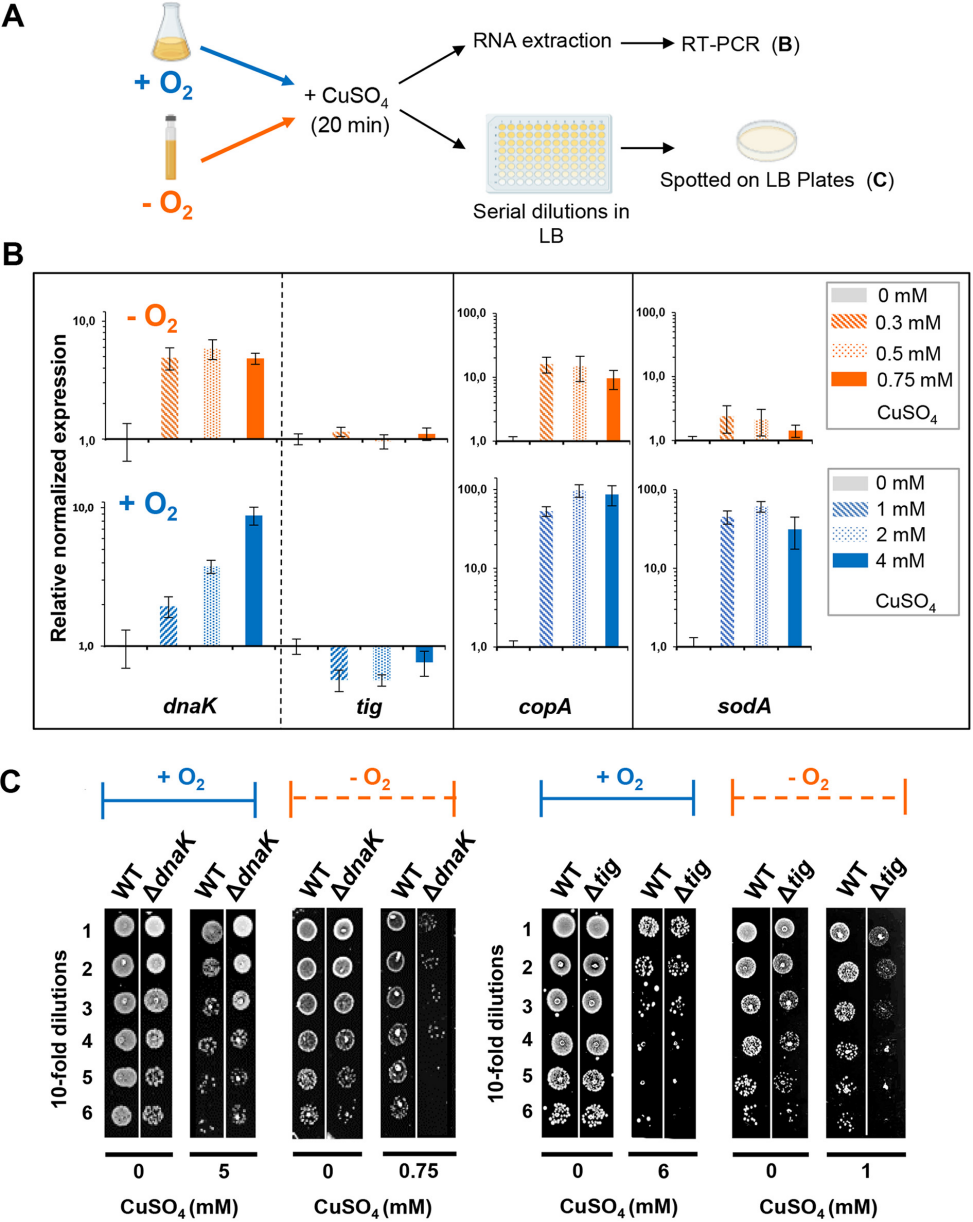
Gene response and role of molecular chaperones after short-term exposure to copper. (A) E. coli culture was exposed to different CuSO_4_ concentrations either anaerobically (orange) or aerobically (blue) for 20 min at 37°C (B) or 30°C (C). The cells were directly centrifuged to extract RNA from the WT strain (B) or serially diluted in LB and spotted on plates (C). In contrast to Fig. 1, no washing step was added because the cells were either quickly centrifuged (B) or diluted in LB, which quenches the excess of copper (C). (B) Quantitative RT-PCR analysis of different sets of genes encoding molecular chaperones (*dnaK* and *tig*) and involved in the heat shock response (*dnaK*), copper homeostasis (*copA*), or the oxidative stress response (*sodA*). The standard deviation is represented by error bars calculated from at least three independent experiments. (C) The WT or the Δ*dnaK* and Δ*tig* mutant strains were stressed and grown under aerobic (blue) or anaerobic (orange) conditions. Plates were incubated at 30°C overnight, and the results are representative of at least three experiments.

Our results showed that both Cu^+^ and Cu^2+^ trigger the aggregation of a large number of proteins. We next monitored the expression level of genes known to be involved in the protection of cells against stress conditions that cause protein misfolding, particularly genes involved in the heat shock response (30). We monitored the expression levels of *dnaK*, *dnaJ*, and *htpG* because the three genes encode different classes of canonical molecular chaperones (Hsp70, Hsp40, and Hsp90, respectively). The expression profiling was conducted in E. coli cells treated with different concentrations of CuSO_4_ for 20 min under either aerobic or anaerobic conditions (Fig. 4A). All three selected heat shock genes were significantly upregulated in cells growing under anaerobic conditions at the lowest Cu concentration tested (0.3 mM) (Fig. 4B; see Fig. S6A in the supplemental material). Under aerobic conditions, induction of these three *hsp* genes reached a significant induction level upon the presence of 4 mM CuSO_4_ in the medium (Fig. 4B; Fig. S6A). As a positive control, we measured the expression levels of *copA* (Fig. 4B) and *cusF* (Fig. S6B) under Cu^+^ and Cu^2+^ stress. Both genes are known to be induced by copper stress and responsible for maintaining copper homeostasis by exporting excess copper into the medium (31, 32). Accordingly, an increase of their expression levels is clearly monitored from a 10- and up to 1,000-fold increase under both stress conditions (Fig. 4B; Fig. S6B). In addition to the heat shock genes, we also monitored the expression levels of *tig*, a constitutively expressed gene that encodes the well-known chaperone trigger factor as well as *cpxP*, an indicator of the envelope stress response (33). Whereas the expression levels of *tig* did not change upon either of the copper treatments (Fig. 4B), *cpxP* was induced upon the addition of the lowest concentration of copper under both aerobic and anerobic conditions (Fig. S6C). Overall, our results revealed that copper treatment triggers the heat shock and envelope stress response in an apparent effort to mitigate misfolding and protein aggregation. To validate our hypothesis that cells growing anaerobically in the presence of copper do not undergo oxidative stress in contrast to aerobically growing cells, we monitored the expression level of the gene *sodA*, encoding an antioxidant superoxide dismutase, known to be upregulated by the SoxR regulator in response to oxidative stress (34). We observed a marked overexpression of *sodA* when cells are exposed to CuSO_4_ under aerobic conditions but not under anaerobic conditions (Fig. 4B). This result supports our conclusion that under anaerobic growth conditions, copper stress does not lead to ROS production. We did an additional experiment to confirm the absence of oxidative stress under our anaerobic growth conditions. We used an E. coli strain lacking the periplasmic disulfide isomerase DsbC, a protein known to rearrange incorrect disulfide bonds in periplasmic proteins. This strain has previously been shown to be highly sensitive toward copper stress under aerobic conditions (also observed in Fig. S7 in the supplemental material), presumably due to its inability to repair oxidative stress-induced nonnative disulfide bonds (35). When we treated the cells with copper under anaerobic conditions, however, the deletion of *dsbC* had no impact on cell survival (Fig. S7), further supporting the conclusion that oxidative stress is not involved in the toxicity of anaerobic copper stress.

10.1128/mbio.03251-21.6 FIG S6 Quantitative RT-PCR analysis of *dnaJ* and *htpG* (A), *cusF* (B), and *cpxP* (C). E. coli cultures were exposed to increasing CuSO_4_ concentrations under either anaerobic (orange) or aerobic (blue) conditions for 20 min at 37°C. Cells were harvested, and gene expression was measured by quantitative RT-PCR as described in Materials and Methods. The standard deviation is represented by error bars calculated from at least three independent experiments. Download FIG S6, JPG file, 0.1 MB. Copyright © 2022 Zuily et al. 2022 Zuily et al. https://creativecommons.org/licenses/by/4.0/ This content is distributed under the terms of the Creative Commons Attribution 4.0 International license.

**FIG 5:**
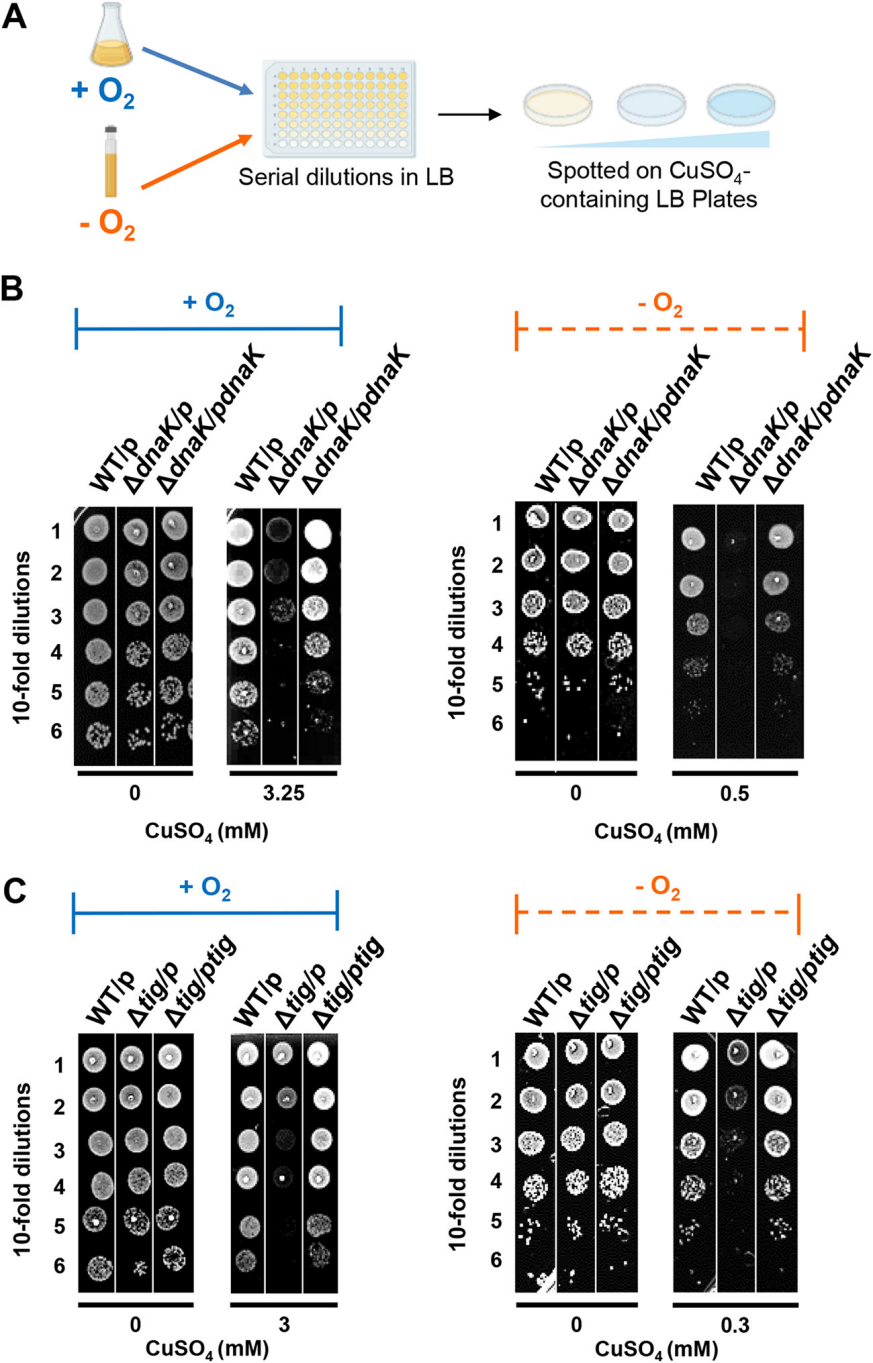
Molecular chaperones protect cells against long-term exposure to copper under both aerobic and anaerobic conditions. (A) Strains were grown at 30°C until they reached an OD of 0.7 under aerobic (in blue) or anaerobic (in orange) conditions. Serial dilutions of these strains were spotted on LB agar supplemented or not with different concentrations of CuSO_4_ and also containing ampicillin and IPTG. Plates were incubated in an aerobic or anaerobic atmosphere overnight at 30°C. (B) The MC4100 WT strain (WT) and the Δ*dnaK*::Cm^r^ (Δ*dnaK*) mutant containing the empty vector pSE380 (p) or the plasmid expressing *dnaK* (*pdnaK*) were grown at 30°C with ampicillin. After following the procedure described for panel A, representative plates are depicted. (C) The MC4100 WT and Δ*tig*::Cm^r^ mutant strain containing the empty vector pSE380 (p) or the plasmid expressing *tig (ptig*) were grown at 30°C and then spotted on LB plates containing copper as described for panel A. Plates are representative of at least three experiments.

10.1128/mbio.03251-21.7 FIG S7 DsbC and DnaK protects cells against copper-induced cell death under aerobic conditions (A) The WT (MC1000) and Δ*dsbC*::Cm^r^ mutant strains were grown at 37°C and then spotted on LB plates containing increasing concentrations of CuSO_4_, as depicted in Fig. 5A. Plates were incubated under aerobic (in blue) or anaerobic (in red) conditions for 20 h at 37°C. Plates are representative of at least three experiments. (B) Growth of E. coli WT (circles) and mutant (Δ*dnaK*) (reversed triangles) strains at 30°C in liquid medium in the presence of increasing concentrations of copper. E. coli cells were grown until they reached an OD_600_ of 0.7 at 30°C under aerobic conditions, and cells were diluted to an OD_600_ of 0.1 in the presence of different CuSO_4_ concentrations (black, 0 mM; red, 2.5 mM; blue, 3.5 mM; purple, 4 mM) at 30°C with shaking using Tecan devices. Absorbance at OD_600_ was measured over time. Several experiments were performed: representative results are shown. Download FIG S7, JPG file, 0.1 MB. Copyright © 2022 Zuily et al. 2022 Zuily et al. https://creativecommons.org/licenses/by/4.0/ This content is distributed under the terms of the Creative Commons Attribution 4.0 International license.

### Molecular chaperones protect E. coli against Cu stress.

Our results raised the question as to the precise role that cytoplasmic molecular chaperones play under Cu^+^ and Cu^2+^ stress in bacteria. The E. coli DnaKJE chaperone machinery assists in protein folding and protects against stress-induced protein unfolding through an ATP-dependent cycle of client protein binding and release (36–38). This chaperone complex has been extensively studied over the years, yet its role under copper stress conditions has not been investigated. We therefore treated E. coli wild-type or Δ*dnaK* strains for 20 min with different concentrations of CuSO_4_ under aerobic or anaerobic conditions and monitored growth on LB agar plates after serial dilutions (Fig. 4C). Under anaerobic conditions, we observed that the Δ*dnaK* strain is highly sensitive to copper stress and does not survive exposure to 0.75 mM CuSO_4_, a concentration that the WT strain tolerates (Fig. 4C). In contrast, under aerobic growth conditions, WT and Δ*dnaK* strains behave very similarly, and both strains significantly grow in the presence of 5 mM CuSO_4_ (Fig. 4C). Similar results were obtained when we tested the Δ*tig* strain, which lacks trigger factor (Fig. 4C). Our results demonstrate a key role of trigger factor and the DnaK system to maintain proteostasis upon short-term exposure to Cu^+^ stress, which leads to widespread protein unfolding and aggregation. They furthermore imply that the toxicity of a short-term treatment with copper under aerobic conditions is independent of protein aggregation and hence cannot be prevented by molecular chaperones. In contrast, when we spotted cells on LB plates containing increasing concentrations of CuSO_4_ (long-term exposure) (Fig. 5A), we observed that the growth of both Δ*dnaK* and Δ*tig* strains is strongly affected under both aerobic and anaerobic conditions (Fig. 5B and C). We also noted that in the presence of copper, the Δ*dnaK* strain grew more slowly than the WT strain in liquid cultures under aerobic conditions (Fig. S7). Ectopic expression of *dnaK* or *tig* restored the copper-dependent growth defect in the respective deletion strains (Fig. 5B and C).

Overall, these results demonstrate that molecular chaperones play central roles in protecting bacteria against exposure to extracellular copper excess.

## DISCUSSION

**FIG 6:**
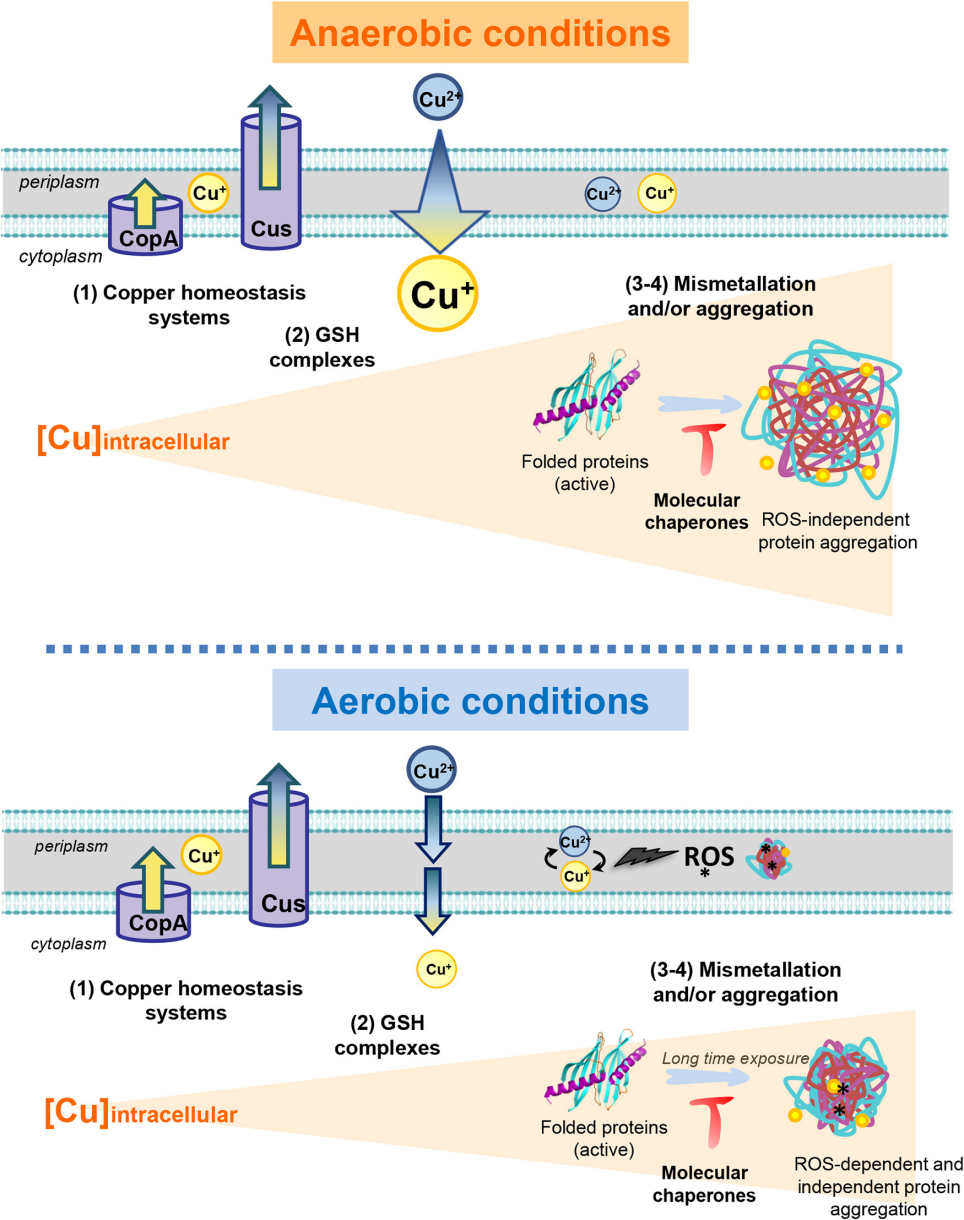
Model of CuSO_4_ effects on protein folding and chaperone function under aerobic or anaerobic growth conditions. Under non-stress conditions, proteins are well folded and active. Exposure of proteins to CuSO_4_ (Cu^2+^) under anaerobic conditions leads to the intracellular accumulation of Cu^+^ (arrow). Depending on the CuSO_4_ concentration and the incubation time, a gradual increase of intracellular copper content will occur (represented by the triangular orange shading). This will first induce the expression of copper homeostasis systems (1st phase). In this figure, only two systems are shown: the CopA and Cus systems. They are both known to export intracellular copper excess. Intracellular cytoplasmic copper will react with GSH (2nd phase), which will limit the accumulation of free intracellular copper. A higher concentration of intracellular copper will result in mismetallation of cytoplasmic proteins (3rd phase), as well as provoke widespread protein unfolding and aggregation (4th phase). Such aggregation is likely due to its ability to coordinate neighboring cysteines and histidines in proteins. Molecular chaperones such as DnaKJE and trigger factor prevent cell death under such conditions by maintaining proteostasis. Under aerobic conditions, ROS will be produced by the Fenton reaction catalyzed by copper, particularly in the periplasmic oxidative environment. These ROS (where the asterisk represents potential oxidative modifications) will react with most macromolecules and cause severe cellular damage. After long-term exposure to copper, copper will not only be involved in the Fenton reaction and/or disulfide stress, but will also induce cytoplasmic reactions similar to those shown under anaerobic conditions (including from the 1st to 4th phases) and will also end up with protein aggregation. Under such growth conditions, molecular chaperones protect cells against this copper-generated stress.

Copper has long been known for its cytotoxicity, and it is now recognized that the innate immune system triggers bacterial death through an increase of copper levels in the phagolysosome (39, 40). Although it has been shown that copper causes cell death due to an increase in periplasmic ROS production via the Fenton reaction and inactivation of proteins by mismetallation (13, 32), other studies have proposed a role for copper as an unfolding agent based on results obtained on purified protein (21). In support of this hypothesis, Wiebelhaus et al. (17) identified partially unfolded proteins in E. coli after copper stress treatment. Here, we demonstrate that copper, in contrast to other metals (such as iron, zinc, or nickel), causes massive global protein aggregation *in vivo* and *in vitro*, an effect that is especially pronounced under anaerobic growth conditions (Fig. 6).

This widespread proteotoxic effect might explain the ability of copper to kill a multitude of organisms ranging from eukaryotic cells to bacteria and viruses. It is noteworthy that hypochlorous acid, the main compound in household bleach and one of the most effective disinfectants known, has been shown to also kill organisms through widespread protein unfolding and aggregation (41). It is this broad reactivity of copper that spurs our research and the search for Cu-based compounds that can counter the rise of antibiotic-resistant strains (9, 17, 42, 43).

### Both copper redox states cause protein aggregation *in vitro*.

Our *in vitro* work demonstrates that both copper redox states trigger protein aggregation, clearly excluding ROS formation as the primary proteotoxic culprit. *In vivo*, however, we observed protein aggregation when E. coli cells were grown under anaerobic conditions in the presence of copper. Analysis of the intracellular copper concentrations revealed that anaerobically grown bacteria accumulate up to 3-fold more copper intracellularly than aerobically grown bacteria, which might be one explanation of the *in vitro* versus *in vivo* discrepancy in our results. Proteomic analysis of the *in vitro-*formed aggregates, however, also revealed some qualitative differences and demonstrated that Cu^+^-sensitive proteins contain on average a higher number of Cys and His residues than Cu^2+^-sensitive proteins. This result is in agreement with the notion that Cu^+^ (like Ag^+^) is a soft Pearson's acid, which exerts a much higher thiophilicity than the intermediate Pearson's acid Cu^2+^ (44). It is likely that Cu^+^ coordination to Cys and His residues in proteins stabilizes potentially nonnative conformations, triggers local unfolding events, and leads to protein aggregation. In contrast, Cu^2+^ might interfere with protein folding by other means, such as its ability to directly oxidize side chains and/or by indirectly causing side chain modifications through ROS production.

### Gradual impact of copper on cells.

A high level of cytoplasmic GSH protects cells from metal-related damage (45–47). Indeed, previous *in vitro* studies showed that Cu^2+^ triggers the aggregation or the inactivation of purified proteins, such as bovine serum albumin or fumarase A, which can be effectively prevented by GSH (13, 21). In addition, the deletion of *gshA* or *gshB* genes renders cells more sensitive to short-term exposure to copper stress under the anaerobic condition. Under the aerobic condition, we did not observe difference in the viability of the WT and Δ*gshA* or Δ*gshB* strains in the presence of copper, most likely because cells might die before any cytoplasmic copper increase. In agreement with our results, a previous report (45) showed, under aerobic conditions, a high sensitivity of the Δ*gshA* and Δ*gshB* strains to copper in E. coli strains with the *copA* gene deleted. Based on our results, we propose that under their conditions, the absence of CopA artificially allows cytoplasmic copper increase, whereas under anaerobic conditions, cytoplasmic copper accumulates without the need to delete *copA*. Although we cannot rule out that some proteins are being protected by GSH against Cu-induced aggregation, our data showed no significant protective effect of GSH when we tested the impact of Cu-GSH complexes on cellular extracts. Moreover, proteins aggregated *in vivo* in the presence of a high level of endogenous GSH. These results suggest that a substantial number of proteins can be sensitive to Cu^+^ treatment even in the presence of intracellular GSH. This finding expands on recent work that suggested a gradual impact of copper on cells, depending on the amount of intracellular copper, the first state, which corresponds to the induction of copper efflux systems to maintain intracellular copper homeostasis, the intermediate state, in which copper is buffered by the GSH (a strain deficient in GSH production is more sensitive to increased intracellular copper), and a third state, in which the ability of GSH to cope with copper is limited by increasing intracellular copper concentrations, subsequently leading to protein mismetallation (48) (Fig. 6). Based on our data, we now propose a fourth phase, where high intracellular copper levels cause nonspecific protein binding followed by protein aggregation (Fig. 6).

### Anaerobic copper stress boosts intracellular copper levels.

Intracellular copper measurements revealed that the oxygenation levels during the copper treatment strongly affected the intracellular copper concentrations (Fig. 6). Consistent with previous reports, we found that under anaerobic conditions, cells treated with CuSO_4_ accumulate significantly higher levels of copper than cells grown under aerobic conditions (49, 50). Although cells are treated with Cu^2+^ (CuSO_4_), intracellular copper is rapidly reduced to Cu^+^, which is therefore the predominant form in the bacterial cytoplasm under all growth conditions (32).

We now consider several possibilities to explain these results. The periplasmic protein CueO, which converts Cu^+^ into Cu^2+^ by reducing oxygen into water, is therefore no longer active under anaerobic conditions (31, 51). Bacteria would thus accumulate periplasmic Cu^+^, which might cross the membrane more easily than the highly charged Cu^2+^ (32). In agreement, Tree et al. (52) observed an increase in intracellular copper in a *cueO* mutant strain. It is also feasible that Cu transport is affected under anaerobic conditions, either by reducing the activity of the Cu-ATPase pump or by other alternative pathways. In any case, our work underscores the importance of measuring the intracellular amount of copper to clarify the reason for cell death.

### Different mechanisms are involved in copper-mediated cell death.

Our finding that the absence of the chaperones’ trigger factor or DnaKJE increases copper toxicity supports our conclusion that both short- and long-term exposure to copper stress under anaerobic conditions cause cell death through an imbalance in proteostasis and increased protein aggregation. In contrast, short-term copper exposure under aerobic conditions does not lead to massive accumulation of intracellular copper and causes cell death, even without significant protein aggregation. The reason for aerobic copper toxicity might be related to the oxidative inactivation of critical periplasmic/membrane proteins. A combination of several factors such as oxidative stress but also protein aggregation could explain the toxicity when cells are exposed for a long time to a lower concentration of copper since we observed a requirement for cytoplasmic molecular chaperones (Δ*dnaK* or Δ*tig* mutant), and periplasmic proteins (Δ*dsbC* mutant).

Proteostasis imbalance induced by copper could explain the broad impact of copper on cells by the inactivation of several pathways. Our mass spectrometry analysis of copper-sensitive proteins *in vitro* highlights numerous pathways that could be affected by copper *in vivo*. For instance, we found that Cu^+^ induces Zur aggregation, which could result in an inability of cells to respond to zinc starvation. In agreement with this result, Kershaw et al. (53) found a decrease in expression of the zur-dependent operon (*znuABC*) in response to an increase in copper concentration. Such cross talk between different metal homeostasis systems has been shown by others (54) and will represent attractive research topics for future studies.

When using copper as an antibacterial agent, several parameters must be taken into consideration: the main redox species, the complexation of the metal, the exposure time, and the concentration, as well as the oxygen level. Depending on these nonexclusive parameters, cells will react differently. On the other hand, their metabolism, growth phase, and/or gene expression represent other parameters to be taken into account in the future in order to overcome or optimize the death of bacteria. For instance, it was previously shown that strains growing on different carbon sources react differently to copper stress (45).

### Cellular protection against copper stress by molecular chaperones.

Protecting cells against proteotoxic agents involves expelling or scavenging the culprits and repairing the damage by refolding proteins and/or preventing their aggregation. We speculated that if the copper-induced death was indeed due to protein aggregation, bacteria lacking molecular chaperones should be more sensitive to copper. Indeed, we were able to demonstrate that cytoplasmic molecular chaperones play a crucial role in protecting bacteria against copper toxicity (Fig. 6). We found that the DnaKJE chaperone machine as well as the trigger factor play a key role in protecting cells against copper stress. Interestingly, other essential metals found in cells (iron, zinc, nickel, etc.) did not induce protein aggregation under the conditions tested, unlike highly toxic nonessential heavy metals (such as arsenic and cadmium), which also trigger protein misfolding and aggregation (55, 56). Although essential for most cells, copper has properties similar to those of highly toxic metals, emphasizing the importance for cells to tightly control the amount as well as the impact of intracellular copper. For these reasons, cellular mechanisms exist to protect the cell against the proteotoxicity of this metal. Indeed, we show in our study that the molecular chaperones tested appear to be active under copper stress, whereas it has been shown that arsenic inhibits chaperone activity (55). Therefore, because the chaperone proteins behave differently in the presence of metals, we can speculate that cells have adapted to survive in the presence of a low concentration of intracellular copper.

Overall, we show that under anaerobic condition (i) intracellular copper accumulates, (ii) copper induces protein aggregation, even in the presence of intracellular GSH, and (iii) major molecular chaperones like DnaKJE and trigger factor protect cells against this threat (Fig. 6). As copper stress reflects an environment often encountered during microbial infection, molecular chaperones could therefore be regarded as potential pharmacological targets. Indeed, inhibition of these molecular chaperones will lower bacterial defense mechanisms and help the immune system to kill invading pathogens. In addition, understanding the molecular mechanism responsible for the Cu^+^/Cu^2+^-mediated cytotoxicity is also of widespread interest in neurodegenerative diseases. Cu^+^/Cu^2+^ has been shown to play a critical role in the progression of diseases, such as Parkinson’s disease, Alzheimer’s disease, or prion disease (57–59). These diseases are associated with protein misfolding and the deposition of aggregated proteins. Our work opens new avenues concerning (i) the development of copper chelating agents that could help prevent Cu-induced cellular damage and (ii) the role molecular chaperones might achieve to protect the cells from this metal.

## MATERIALS AND METHODS

### Bacterial strains and culture conditions.

The Escherichia coli strains and the plasmids used in this study are listed in Table S3 in the supplemental material. Cells were grown either under aerobic conditions in LB medium with shaking at 160 rpm or under anaerobic conditions in LB supplemented with 20 mM MOPS (morpholinepropanesulfonic acid) and 45 mM glucose in Hungate culture tubes in order to maintain an anaerobic environment, without agitation.

10.1128/mbio.03251-21.10 TABLE S3 Strains and plasmids used in this study. Download Table S3, DOCX file, 0.01 MB. Copyright © 2022 Zuily et al. 2022 Zuily et al. https://creativecommons.org/licenses/by/4.0/ This content is distributed under the terms of the Creative Commons Attribution 4.0 International license.

### Metal solutions.

The following metal solutions were used. For Cu^2+^, CuSO_4_ (Sigma, no. C3036) was resuspended in H_2_O. For Cu^+^, tetrakis(acetonitrile) copper(I) hexafluorophosphate (Sigma, no. 346276) was resuspended in ultrapure water with 10% acetonitrile (ACN) (Sigma, no. 271004), stored, and used in the anaerobic chamber. Acetonitrile forms complexes with Cu^+^, which stabilizes it in water, preventing its disproportionation in Cu^0^ and Cu^2+^ (60). As a control, ultrapure water with 10% acetonitrile was used. Cu-GSH is a mixture of 10 mM CuSO_4_ with 100 mM freshly prepared GSH (Sigma, no. G4251) used in the anaerobic chamber. This mixture was added to the assay mixture to obtain a final concentration of 500 μM CuSO_4_ and 5 mM GSH (when mentioned in the experimental setup, 100 μM CuSO_4_ was also prepared with another CU-GSH mixture in order to get a final concentration of 100 μM CuSO_4_ in a final concentration of 5 mM GSH). For Ag^+^, AgNO_3_ (Normapur, no. 21572) in H_2_O was used under anaerobic conditions and stored in the dark. FeSO_4_, ZnSO_4_, NiSO_4_, CoSO_4_, and MnSO_4_ were dissolved in H_2_O and used under aerobic conditions. All the experiments in anaerobic conditions were carried out in a Jacomex glove box under a nitrogen atmosphere.

### Cell survival after short-term exposure to copper.

E. coli cells were grown at 30 or 37°C under anaerobic or aerobic conditions until they reached an optical density at 600 nm (OD_600_) of 0.7. Cells were then exposed to increasing CuSO_4_ concentrations for 20 min. Cells were either directly spotted on plates as described below (see also Fig. 4A), or cells were then harvested by centrifugation at 4,000 rpm for 10 min at 4°C and washed (see the scheme in Fig. 1A). For the anaerobic samples, the following washing steps were performed with buffers degassed with argon and in a glove box to maintain an anaerobic atmosphere. The cell pellets were resuspended with 10 mL of phosphate-buffered saline (PBS) buffer (137 mM NaCl, 2.7 mM KCl, 10 mM Na_2_HPO_4_, 1.8 mM KH_2_PO_4_) containing 50 mM EDTA. These steps (centrifugation and the washing step) were repeated three times. Three microliters of 10-fold serial dilutions was spotted on LB agar plates under aerobic conditions or on plates supplemented with 20 mM MOPS and 45 mM glucose under anaerobic conditions. For O_2_-deprived conditions, the plates in the glove box were directly transferred in anaerobic jars with an anaerobic filter (Anaerocult A). The plates were placed either at 30 or 37°C for 36 h under anaerobic conditions or for 24 h under aerobic conditions.

### Survival test after long-term exposure to copper.

The E. coli strains mentioned in Table S3 (WT and Δ*dnaK*::Cm^r^ or Δ*tig*::Cm^r^ mutant) were freshly transformed when needed with plasmid pSE380, pSE380(*dnaK*), or pSE380(*tig*), respectively. Cells were grown at 30°C in LB medium supplemented with glucose (45 mM), MOPS (20 mM), and, when necessary, with ampicillin (1 mM) until the OD_600_ reached 0.7 under either aerobic or in anaerobic conditions. Ten-fold serial dilutions were prepared, and 3 μL was spotted on LB agar plates containing increasing CuSO_4_ concentrations supplemented with 1 mM ampicillin (when cells contained a plasmid), with or without isopropyl-β-d-thiogalactopyranoside (IPTG) (0 to 5 μM). The plates were incubated in an aerobic or anaerobic atmosphere at 30 or 37°C.

The E. coli strains mentioned in Table S3 (MC4100 WT and Δ*dnaK*::Cm^r^ mutant) were grown at 30°C in LB liquid medium under aerobic conditions until an OD_600_ of 0.7 was reached. The cells were diluted to a final OD_600_ of 0.1 and were incubated with increasing concentrations of CuSO_4_. The growth of these strains was followed by measuring the absorbance at 600 for almost 7 h.

### Cu quantification by ICP-OES.

E. coli cells were stressed and washed as described in the section “Cell survival after short-term exposure to copper.” After the third wash, each pellet was resuspended in 500 μL of PBS buffer and 500 μL of 69% nitric acid. The samples were boiled for 40 min, and 4 mL of 3% nitric acid was added to each sample. Copper analyses were performed on an ICAP 6000 series optical emission spectrometer (Thermo Scientific). Serial dilutions of copper standard solution were used to calibrate the inductively coupled plasma optical emission spectrometry (ICP-OES) system.

### Purification of protein aggregates from whole cells.

E. coli cells were exposed to stress under anaerobic or aerobic conditions and then washed as described in “Cell survival after short-term exposure to copper.” Note that the following steps were performed under either aerobic or anaerobic conditions (glove box). After the washing steps, each pellet was resuspended in 120 μL of buffer A (10 mM KH_2_PO_4_ [pH 6.5], 1 mM EDTA, 20% [wt/vol] sucrose, 1 mg/mL lysozyme) and incubated for 30 min in ice. A total of 1,080 μL of buffer B (10 mM KH_2_PO_4_ [pH 6.5], 1 mM EDTA) was added to the cells, which were subsequently lysed by sonication, using either a Branson Sonifier 450 (50% duty, level 2, 10 cycles) for aerobic samples or the Ultrasonic processor UP100H (Hielscher) for samples in the anaerobic glove box. After lysis, the cells were centrifuged at 4,000 rpm for 15 min at 4°C to remove unbroken cells. To isolate the insoluble cellular fraction (containing membrane and aggregated proteins), centrifugation at 13,000 rpm for 35 min at 4°C was performed. The pellets were resuspended in 1 mL of buffer B by sonication and centrifuged at 13,000 rpm for 25 min at 4°C. The pellets were resuspended in 960 μL of buffer B by brief sonication, and 240 μL of 10% (vol/vol) NP-40 was added to solubilize the membrane proteins. After homogenization, centrifugation at 13,000 rpm for 35 min at 4°C was performed to isolate the aggregated proteins. This washing and sonication steps were repeated twice to remove most of the membrane proteins, which for an unknown reason, can sometimes be detected in all samples. The pellets were suspended in 60 μL of 6 M urea, then loaded on SDS-PAGE to visualize aggregated proteins. The gels were stained with Coomassie protein stain (Instant Blue).

### Purification of protein aggregates from cell lysate.

First, cell extracts were prepared by the following steps. E. coli cells were grown in LB medium under aerobic conditions until they reached an OD_600_ of 0.6. (Note that the following steps were performed under either aerobic or anaerobic conditions [glove box].) Cells were then harvested by centrifugation at 4,000 rpm for 10 min at 4°C. Cells were resuspended in buffer C (40 mM MOPS, 0.2 M KCl [pH 7.5]) and lysed (Ultrasonic processor UP100H [Hielscher], 2 cycles, 4°C, 160,000 Pa). After ultracentrifugation (45,000 rpm, 1 h 30 min), the protein concentration of the supernatant containing the soluble proteins was determined by bicinchoninic acid (BCA) assay (Sigma), and samples were frozen at −80°C. These cell extracts were then incubated with different stressed agents, and aggregated proteins were isolated as described below. The cell extracts (1 mg/mL) were incubated with or without 100 or 500 μM Cu^2+^ (under aerobic conditions), Cu^+^, Cu-GSH, or Ag^+^ (under anaerobic conditions) at 30°C during 30 min. The aggregates (A) and the soluble proteins (S) were separated by centrifugation (11,000 rpm, 40 min). The proteins from the supernatant were precipitated with trichloroacetic acid (10% [vol/vol] final concentration). Both pellets were resuspended in 8 M urea and analyzed by 12% SDS–PAGE or by mass spectrometry.

### Metal-induced protein aggregation assays.

Citrate synthase (CS) (Sigma), luciferase (Promega), or purified E. coli EF-Tu was diluted into 40 mM MOPS (pH 7.5) at 30°C to a final concentration of 2 μM in the absence or presence of the indicated ratio of Cu^2+^ (under aerobic conditions) or Cu^+^ (under anaerobic conditions). Light scattering at 360 nm was followed using a Cary spectrophotometer for 15 min at 30°C. Alternatively, the samples were taken after 30 min of incubation at 30°C, and protein aggregates were separated from the soluble fraction by centrifugation (11,000 rpm, 40 min) and analyzed by 12% SDS–PAGE.

### Spectroscopic measurements.

Far-UV circular dichroism spectroscopy of 2.5 μM CS was recorded in 40 mM KH_2_PO_4_ (pH 7.5) using a Jasco-815 spectropolarimeter at 25°C. When indicated, a 4-, 8-, 20-, 40-, or 160-fold molar excess of Cu^+^ was added under anaerobic conditions using rubber-sealed cuvettes. All spectra were buffer corrected.

### Citrate synthase activity assay.

The activity of CS was determined according to the method described by Jakob et al. (61). A 0.15 μM concentration of citrate synthase was incubated with 0, 4, 8, 20, 40, 80, or 160 molar equivalents of Cu^2+^ or Cu^+^ under aerobic or anaerobic conditions, respectively, at 25°C. After 30 min of incubation, the activity of CS was determined. The activity of CS in the absence of copper was set to 100%.

### Sample preparation for mass spectrometry.

The protein pellets (also called “A” for “aggregates”) obtained as described in the section “Purification of protein aggregates from cell lysate” were resuspended in 100 μL of a mixture of 6 M urea, 50 mM Tris-HCl (pH 8), and 3 mM dithiothreitol (DTT) and incubated for 2 h at 37°C. After cooling and centrifugation, the thiol groups were alkylated by incubation with 0.1 M iodoacetamide in the dark for 1 h at 350 rpm at 25°C. Then, the urea buffer was diluted with the digestion buffer (50 mM Tris buffer [pH 8] plus 10% acetonitrile) to 1 M, and the digestion was carried out overnight at 37°C at 350 rpm using 1.5 μg of Trypsin Gold of mass spectrometry grade (Promega). The peptide concentration was determined, after which the peptides were loaded onto C_18_, in-house, stage tips in equal amounts as described in reference 62. Three biological replicates for each of the four conditions were subsequently analyzed by liquid chromatography mass spectrometry.

### Nano-LC-MS/MS analysis.

For nanoscale liquid chromatography-tandem mass spectrometry (nano-LC-MS/MS), the peptides (1.5 μg) of each sample were injected and washed with 4% acetonitrile and 0.1% formic acid for 45 min at a flow rate of 300 nL/min and separated on a C_18_ reverse-phase column coupled to the nano-electrospray EASY-spray device (PepMap, 75 mm by 50 cm; Thermo Scientific) using an Dionex Nano-HPLC (i.e., high-performance liquid chromatography) system (Thermo Scientific) coupled online to an Orbitrap mass spectrometer, Q Exactive HF (Thermo Scientific). The following linear gradient was applied with a flow rate of 150 nL/min at 45°C: from 1% to 28% in 90 min, from 28% to 50% in 17 min, and from 50% to 80% in 10 min, followed by being held at 80% for an additional 13 min and then equilibrated at 1% for 20 min (solvent A is 0.1% formic acid, and solvent B is 80% acetonitrile plus 0.1% formic acid). Additional column washes with 80% ACN for 40 min were carried out between each sample run to avoid potential carryover of the peptides. The Q Exactive HF was operated in a data-dependent mode. The survey scan range was set to 300 to 1,650 *m*/*z*, with a resolution of 60,000 at *m*/*z*. Up to the 15 most abundant isotope patterns with a charge of ≥2 were subjected to higher-energy collisional dissociation with a normalized collision energy of 27, an isolation window of 1.6 *m*/*z*, and a resolution of 15,000 at *m*/*z*. To limit repeated sequencing, dynamic exclusion of sequenced peptides was set to 20 s. Thresholds for ion injection time and ion target value were set to 20 ms and 3 × 10^6^ for the survey scans and to 25 ms and 10^5^ for the tandem mass spectrometry (MS/MS) scans. Only ions with the “peptide preferable” profile were analyzed for MS/MS. Data were acquired using Xcalibur software (Thermo Scientific). The raw mass spectrometry data were uploaded to the PRIDE public site (accession no. PXD019288).

### Data analysis and statistics for mass spectrometry.

For protein identification and quantification, we used the MaxQuant software version 1.6.3.3 (25). We used Andromeda search incorporated into MaxQuant to search MS/MS spectra against the UniProtKB database of the E. coli K-12 proteome (2018 Uniprot release). The enzyme specificity was set to trypsin, allowing cleavage N terminal to proline and a maximum of two miscleavages. Peptides had to have a minimum length of seven amino acids to be considered for identification. Carbamidomethylation was set as a fixed modification, and methionine oxidation was set as a variable modification. A false-discovery rate (FDR) of 0.05 was applied at the peptide and protein levels. An initial precursor mass deviation until 4.5 ppm and fragment mass deviation until 20 ppm were allowed. Only proteins identified by more than two peptides were considered (Table S1). To quantify changes in protein expression, we utilized LFQ using the MaxQuant default parameters (25). The Perseus software was used for statistical analysis, defining significantly different protein profiles as well as for visualization (63). For functional annotation analysis, the DAVID web server (https://david.ncifcrf.gov) was used. Statistical analysis of the sequence features (amino acid propensity, hydrophobicity, net charge, and others) was done using in-house scripts. The hydrophobicity and instability were calculated using the GRAVY index scale (64) and as described by Guruprasad et al. (65), respectively. The frequencies of the disorder and amyloid fragments were calculated using the PASTA 2.0 (26). The sequence features were normalized to the length of a given sequence.

### Quantitative real-time RT-PCR.

For quantitative real-time reverse transcription-PCR (RT-PCR), the E. coli WT MC4100 strain was cultivated at 37°C under either aerobic or anaerobic conditions until the cells reached an OD_600_ of 0.7. The cultures were incubated with 0, 1, 2, or 4 mM CuSO_4_ and 0, 0.3, 0.5, or 0.75 mM CuSO_4_, under aerobic or anaerobic conditions, respectively, for 20 min at 37°C without agitation. The cells (around 2 × 10^9^ cells) were centrifuged at 8,300 rpm for 15 min at 4°C, frozen with liquid nitrogen, and stored at −80°C. Total RNAs were extracted from cells using a Maxwell 16 LEV microRNA (miRNA) tissue kit (Promega) and quantified by spectrophotometry at 260 nm (NanoDrop 1000; Thermo Fisher Scientific). One microgram of total RNA and 0.5 μg of primers (Promega) were used with GoScript reverse transcriptase (Promega) to perform DNAc synthesis. To determine the amplification kinetic, the fluorescence of the EvaGreen dye incorporated into the PCR product was measured at the end of each cycle using SoFast EvaGreen Supermix 2× kit (Bio-Rad, France). The gene *gapA* was used as reference for normalization. For each point, a technical triplicate was performed, and the amplification efficiency for each primer pair was approximately 80 to 100%.
