# Supplementary figures and images for: Copper Induces Protein Aggregation, a Toxic Process Compensated by Molecular Chaperones
Source: mBio. 2022 Mar 15;13(2):e03251-21. doi: 10.1128/mbio.03251-21 (PMC9040851; doi:10.1128/mbio.03251-21)

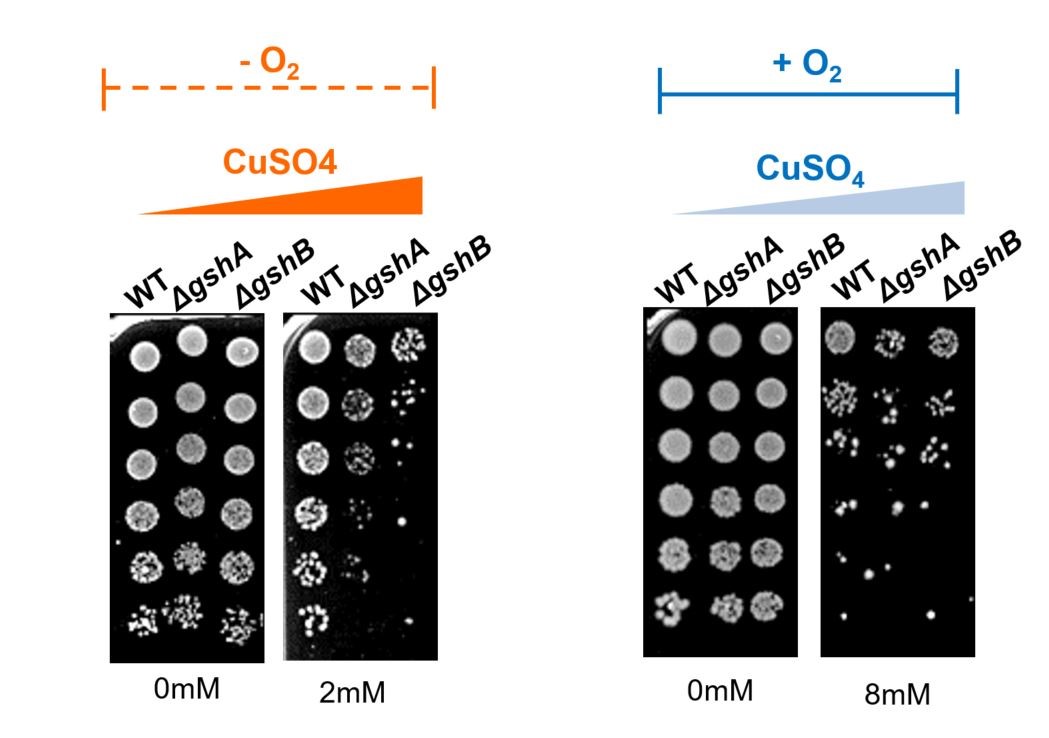

Supplement: FIG S1 [file mbio.03251-21-sf001.jpg]

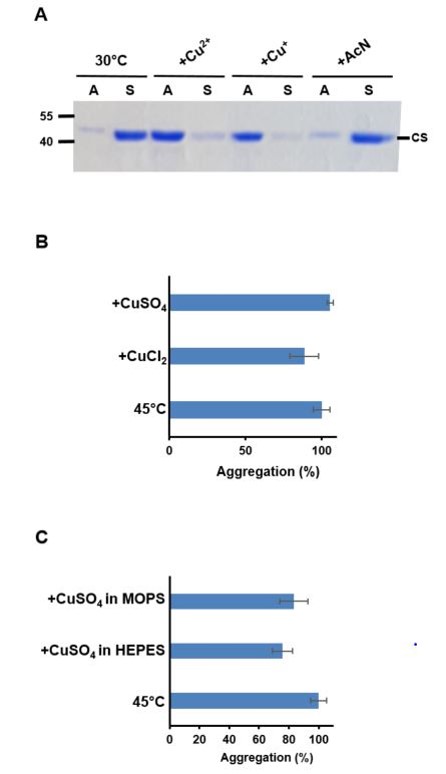

Supplement: FIG S2 [file mbio.03251-21-sf002.jpg]

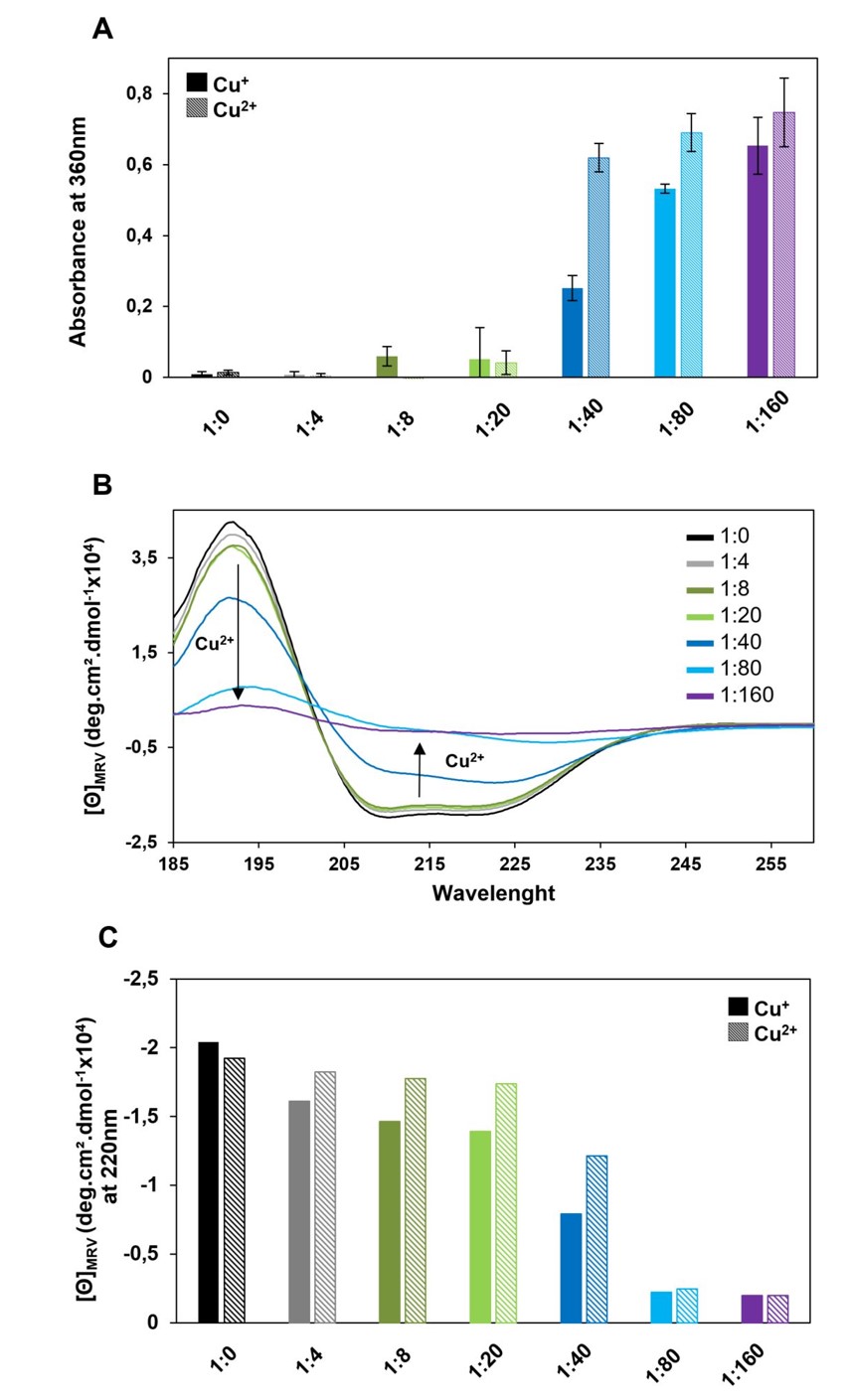

Supplement: FIG S3 [file mbio.03251-21-sf003.jpg]

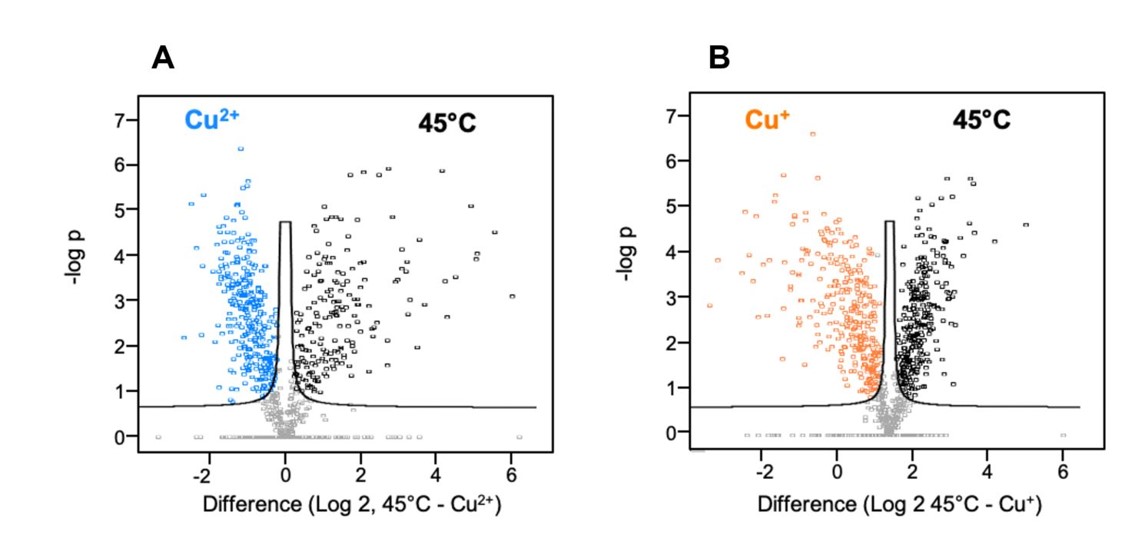

Supplement: FIG S4 [file mbio.03251-21-sf004.jpg]

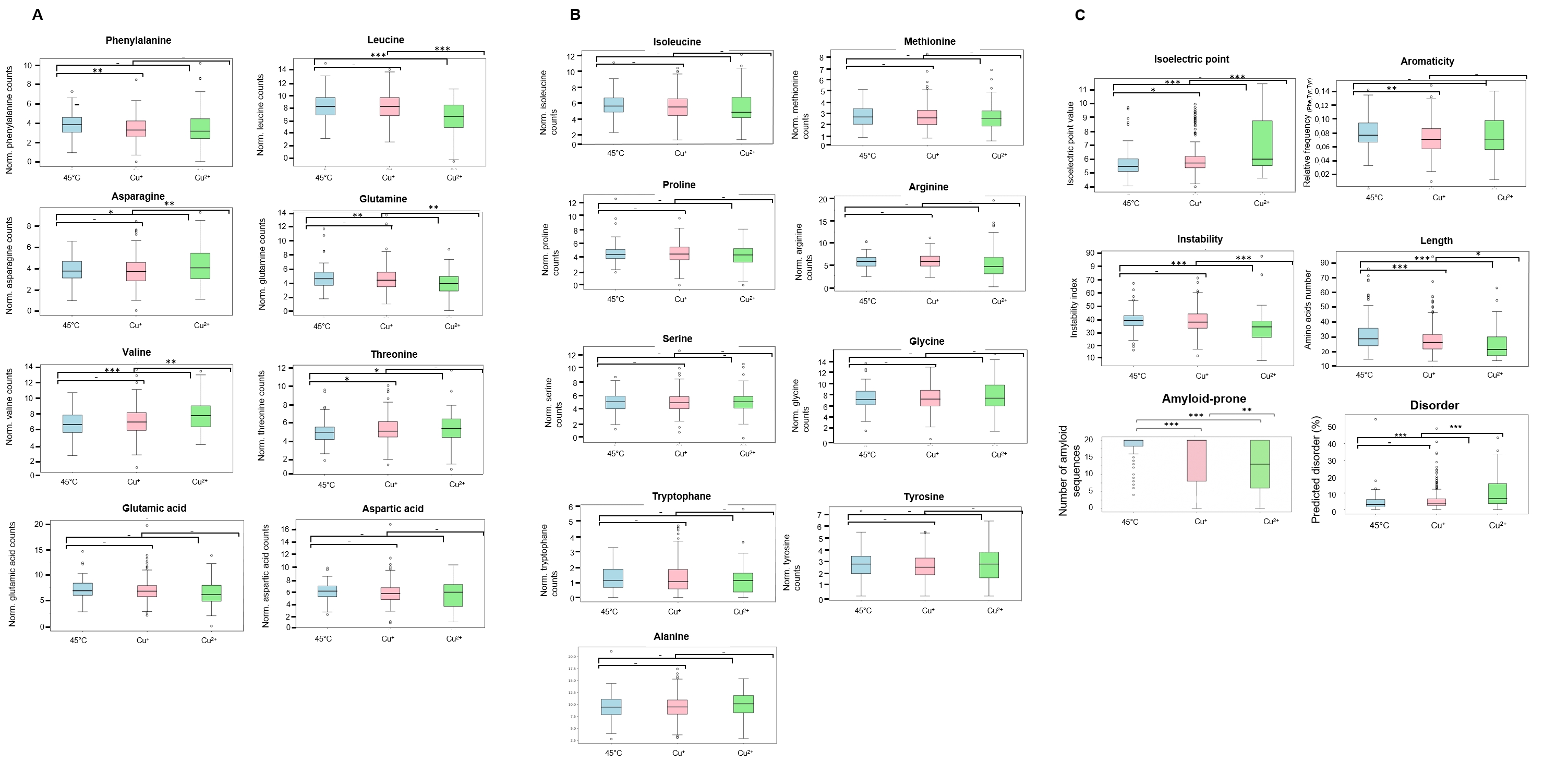

Supplement: FIG S5 [file mbio.03251-21-sf005.jpg]

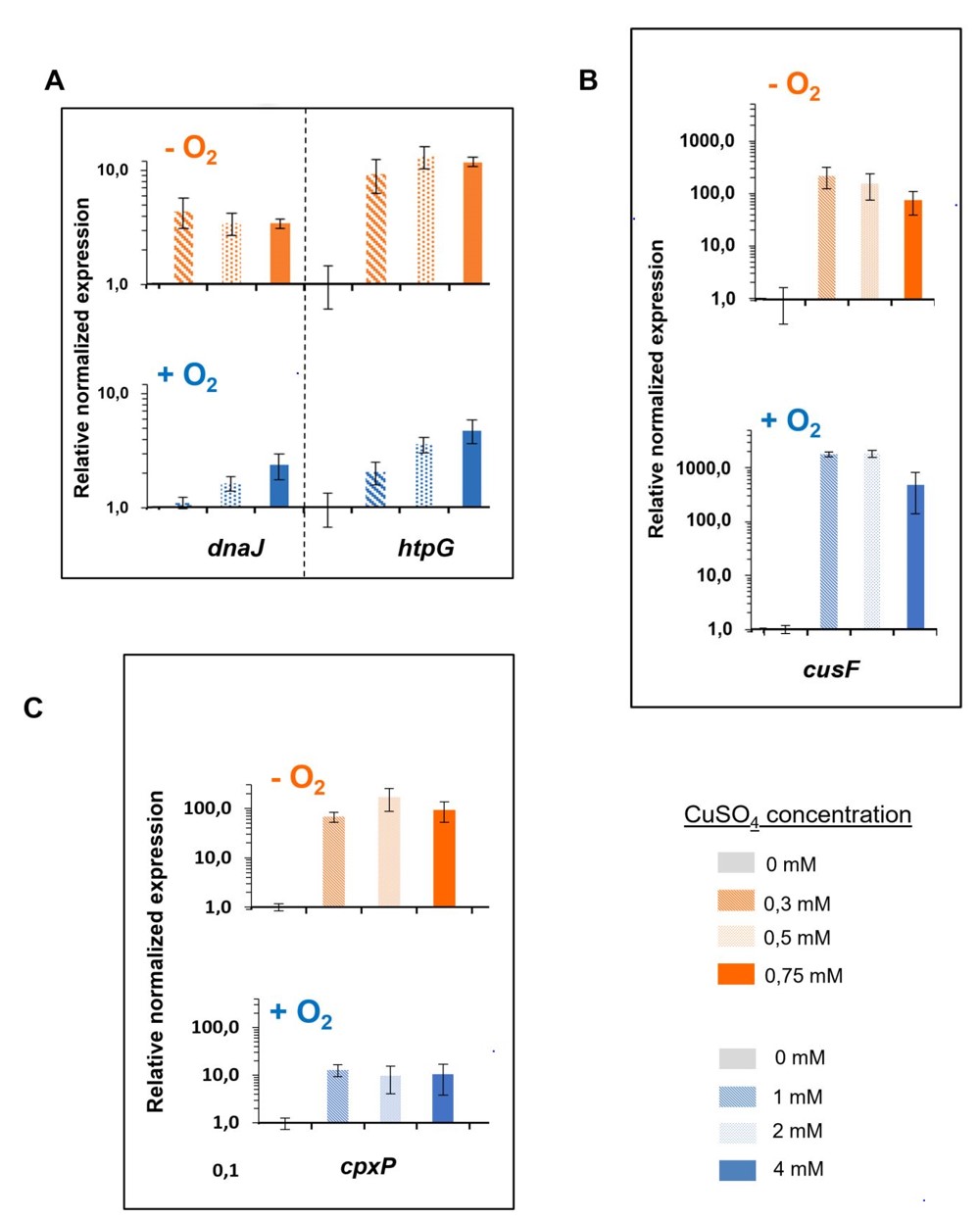

Supplement: FIG S6 [file mbio.03251-21-sf006.jpg]

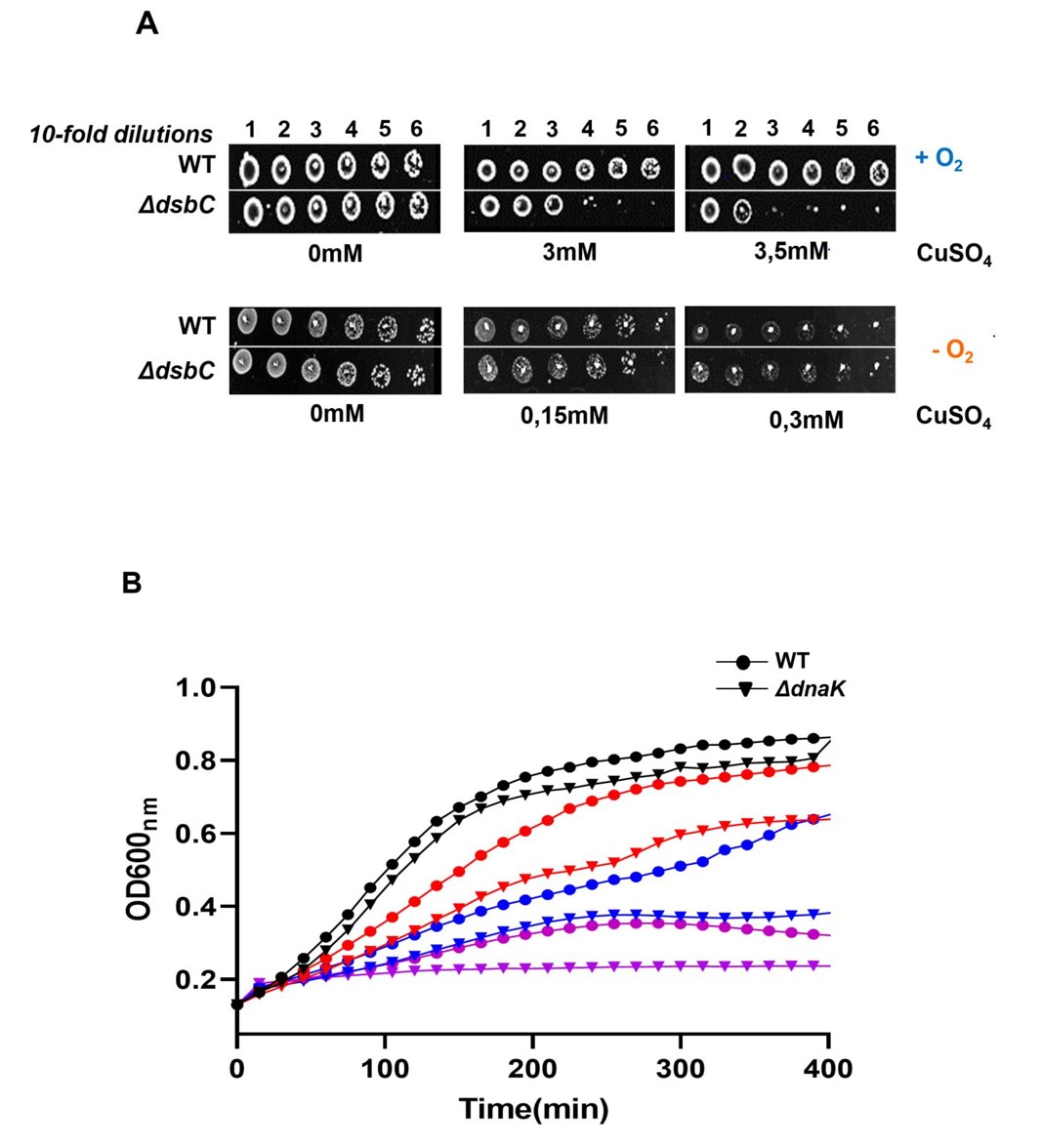

Supplement: FIG S7 [file mbio.03251-21-sf007.jpg]
