# Supplementary material for: Copper Induces Protein Aggregation, a Toxic Process Compensated by Molecular Chaperones
Source: mBio. 2022 Mar 15;13(2):e03251-21. doi: 10.1128/mbio.03251-21 (PMC9040851; doi:10.1128/mbio.03251-21)
Supplement: TABLE S2 [file mbio.03251-21-st002.docx]

**Supplemental Table 2A**

| Treatment | Function | Abundance (%) | P-value |
| --- | --- | --- | --- |
| Heat (45^°^C) | Metal binding | 30 | 3.50E-08 |
|  | Metabolic pathways | 34 | 5.20E-05 |
|  | Nucleotide binding | 26 | 4.60E-03 |
|  | Magnesium binding | 17 | 3.40E-05 |
|  | Oxidoreductase | 15 | 9.60E-05 |
|  | Amino acid biosynthesis | 10 | 2.30E-02 |
|  | NADP binding proteins | 8 | 4.00E-04 |
| Cu^+^ | Metabolic pathways | 38 | 1.20E-10 |
|  | Metal binding | 26 | 9.50E-07 |
|  | Transferase | 22 | 5.70E-05 |
|  | Nucleotide binding | 18 | 2.90E-03 |
|  | Biosynthesis of secondary metabolites | 17 | 1.20E-04 |
|  | Hydrolase | 17 | 3.60E-03 |
|  | Oxidoreductase | 16 | 2.50E-05 |
|  | Magnesium binding | 15 | 3.20E-08 |
|  | Zn binding | 10 | 5.10E-05 |
|  | NADP binding proteins | 9 | 8.80E-07 |
| Cu^2+^ | Ribosomal proteins | 12 | 5.60E-06 |
|  | Disulfide bond | 9 | 3.60E-03 |
|  | Carbon metabolisms | 9 | 5.60E-02 |
|  | Nucleic acid binding | 8 | 7.50E-04 |

Functional annotation of the proteins within subset of either 45°C or Cu^+^ or Cu^2+^ treated cells. Functional annotation was defined by DAVID 6.8 (<https://david.ncifcrf.gov/>).

**Supplemental Table 2B**

| Feature | 45  mean | 45 std | Cu1  mean | Cu1  std | Cu2  mean | Cu2  std | 45 vs Cu1  p-value | 45 vs Cu2  p-value | Cu1 vs Cu2  P-value |
| --- | --- | --- | --- | --- | --- | --- | --- | --- | --- |
| A | 9.50 | 2.60 | 9.51 | 2.45 | 10.05 | 2.57 | 9.92E-01 | 1.75E-01 | 9.08E-02 |
| **C** | **0.98** | **0.61** | **1.43** | **0.79** | **0.68** | **0.67** | **1.07E-06** | **2.80E-03** | **4.05E-13** |
| D | 5.99 | 1.34 | 5.79 | 1.59 | 5.66 | 2.14 | 2.75E-01 | 2.22E-01 | 5.54E-01 |
| E | 7.15 | 1.90 | 6.99 | 1.90 | 6.57 | 2.64 | 4.68E-01 | 9.93E-02 | 1.22E-01 |
| **F** | 3.89 | 1.17 | 3.40 | 1.24 | 3.58 | 1.87 | **1.05E-03** | 1.89E-01 | 3.40E-01 |
| G | 7.29 | 2.04 | 7.23 | 2.04 | 7.66 | 2.77 | 8.01E-01 | 3.28E-01 | 1.38E-01 |
| **H** | **2.18** | **1.01** | **2.77** | **1.14** | **1.84** | **1.17** | **9.51E-06** | 4.63E-02 | **7.78E-10** |
| I | 5.79 | 1.44 | 5.52 | 1.53 | 5.50 | 2.07 | 1.51E-01 | 2.98E-01 | 9.14E-01 |
| **K** | 4.52 | 1.58 | 4.63 | 1.69 | 7.31 | 2.71 | 5.64E-01 | **2.61E-14** | **4.49E-23** |
| **L** | 10.20 | 2.18 | 10.20 | 2.13 | 8.37 | 2.52 | 9.87E-01 | **1.14E-06** | **4.92E-10** |
| M | 2.69 | 0.94 | 2.68 | 1.04 | 2.66 | 1.14 | 9.30E-01 | 8.66E-01 | 9.05E-01 |
| **N** | 3.80 | 1.22 | 3.73 | 1.32 | 4.27 | 1.74 | 6.54E-01 | **4.47E-02** | **3.90E-03** |
| P | 4.64 | 1.52 | 4.57 | 1.48 | 4.23 | 1.63 | 6.77E-01 | 8.94E-02 | 8.29E-02 |
| **Q** | 4.60 | 1.65 | 4.49 | 1.64 | 3.88 | 1.68 | 5.90E-01 | **5.57E-03** | **3.97E-03** |
| R | 5.84 | 1.55 | 5.77 | 1.59 | 5.38 | 3.74 | 7.20E-01 | 2.89E-01 | 1.80E-01 |
| S | 5.08 | 1.39 | 5.08 | 1.40 | 5.21 | 1.66 | 9.72E-01 | 5.63E-01 | 4.91E-01 |
| T | 4.92 | 1.30 | 5.23 | 1.30 | 5.40 | 1.67 | 4.37E-02 | 3.53E-02 | 3.38E-01 |
| **V** | 6.86 | 1.65 | 7.09 | 1.75 | 7.86 | 1.99 | 2.74E-01 | **5.05E-04** | **1.12E-03** |
| W | 1.25 | 0.79 | 1.27 | 0.99 | 1.15 | 0.99 | 8.80E-01 | 4.40E-01 | 3.37E-01 |
| Y | 2.83 | 1.18 | 2.60 | 1.14 | 2.74 | 1.43 | 1.02E-01 | 6.81E-01 | 3.57E-01 |
| **Net charge** | -2.74 | 2.49 | -2.32 | 2.64 | 0.37 | 5.39 | 1.80E-01 | **2.01E-06** | **3.35E-09** |
| **Aromaticity** | 0.08 | 0.02 | 0.07 | 0.02 | 0.07 | 0.03 | **7.33E-03** | 1.88E-01 | 5.21E-01 |
| **Instability** | 38.56 | 8.19 | 37.88 | 8.80 | 32.85 | 12.41 | 5.16E-01 | **4.66E-04** | **7.12E-05** |
| **Length** | 445 | 256 | 353 | 190 | 293 | 204 | **2.79E-04** | **4.39E-05** | 1.66E-02 |
| Hydrophobicity | -0.20 | 0.22 | -0.20 | 0.21 | -0.28 | 0.35 | 8.68E-01 | 6.85E-02 | 1.53E-02 |
| **Isoelectric point** | 5.69 | 1.01 | 5.96 | 1.08 | 6.91 | 2.01 | 3.10E-02 | **9.32E-07** | **8.57E-08** |
| **Amyloid propensity** | 17.89 | 4.09 | 14.59 | 7.24 | 12.56 | 7.52 | **5.08E-05** | **3.94E-08** | 3.16E-02 |
| **Disorder propensity** | 5.12 | 8.22 | 5.90 | 6.33 | 11.02 | 10.19 | 3.52E-01 | **6.13E-05** | **1.22E-07** |
| Predicted a-helix regions | 45.36 | 14.07 | 42.77 | 14.06 | 40.50 | 19.76 | 1.31E-01 | 6.75E-02 | 2.56E-01 |
| Predicted b-strand regions | 14.75 | 7.59 | 16.37 | 8.76 | 18.29 | 13.23 | 1.18E-01 | 3.36E-02 | 1.35E-01 |
| Predicted coil regions | 39.88 | 8.01 | 40.94 | 7.08 | 41.21 | 9.44 | 2.35E-01 | 3.29E-01 | 7.85E-01 |

The p-values for T-student test were calculated using two tail similar variance.
