# Supplementary material for: Copper Induces Protein Aggregation, a Toxic Process Compensated by Molecular Chaperones
Source: mBio. 2022 Mar 15;13(2):e03251-21. doi: 10.1128/mbio.03251-21 (PMC9040851; doi:10.1128/mbio.03251-21)
Supplement: TABLE S3 [file mbio.03251-21-st003.docx]

**Supplemental Table 3**

| **Strains** | **Relevant Genotype, plasmid** |
| --- | --- |
| LZ5 | MC4100 *ΔhslO*::Cm^R^ |
| NLT1 | MC4100 WT |
| NLT2 | MC4100 *Δtig*::Cm^R^ |
| NLT14 | MC4100 *ΔdnaK*::Cm^R^ |
| NL44 | MC4100 WT, pNL1 |
| NL99 | MC4100 *Δtig*::Cm^R^, pNL1 |
| NL66 | MC4100 *Δtig*::Cm^R^, pNL6 |
| NL52 | MC4100 *ΔdnaK*::Cm^R^, pNL1 |
| NL54 | MC4100 *ΔdnaK*::Cm^R^, pNL5 |
| BW25113 | KEIO WT |
| JW2663 | KEIO *ΔgshA*::Kan^R^ |
| JW2914 | KEIO *ΔgshB*::Kan^R^ |
| JP114 | ER1821 WT |
| JP539 | JPP14 *ΔdsbC*::Cm^R^ |
|  |  |
| **Plasmids** | **Gene, Resistance** |
| pNL1 | pSE380(*empty*) (Amp) |
| pNL5 | pSE380(*dnaK*) (Amp) |
| pNL6 | pSE380(*tig*) (Amp) |
